# Multi-kingdom cervical microbiome structure in health and dysbiosis: a cross-sectional study from Kazakhstan

**DOI:** 10.3389/fmicb.2026.1836889

**Published:** 2026-07-27

**Authors:** Aidana Rakhmankulova, Samat Kozhakhmetov, Artur Kovenskiy, Nurislam Mukhanbetzhanov, Nurlubek Katkenov, Zharkyn Jarmukhanov, Milan Terzic, Gauri Bapayeva, Talshyn Ukybassova, Gulzhanat Aimagambetova, Yevgeniy Kim, Berik Primbetov, Balkenzhe Imankulova, Kuralay Kongrtay, Nazira Kadroldinova, Makhabbat Galym, Sanimkul Makhambetova, Kadisha Nurgaliyeva, Zhanar Abdiyeva, Zhanar Zhumakanova, Balnur Smagulova, Elizaveta Vinogradova, Nazira Kamzayeva, Almagul Kushugulova

**Affiliations:** 1 Department of Biomedical Sciences, School of Medicine, Nazarbayev University, Astana, Kazakhstan; 2 Laboratory of Microbiome, Center for Life Sciences, National Laboratory Astana, Nazarbayev University, Astana, Kazakhstan; 3 Clinical Academic Department of Women’s Health, Corporate Fund “University Medical Center, ” Astana, Kazakhstan; 4 Department of Surgery, School of Medicine, Nazarbayev University, Astana, Kazakhstan; 5 Department of Obstetrics and Gynecology #1, NJSC “Astana Medical University”, Astana, Kazakhstan; 6 Interdisciplinary Sports Research, Center for Genetics and Life Sciences, Sirius University of Science and Technology, Sochi, Russia; 7 Corporate Fund “University Medical Center”, Nazarbayev University, Astana, Kazakhstan; 8 Department of Normal Physiology, Sechenov First Moscow State Medical University, Moscow, Russia

**Keywords:** *Candida*, cervical microbiome, cervicovaginal dysbiosis, community state types (CST), *Gardnerella*, HPV-negative, *Lactobacillus*, multi-kingdom microbiota

## Abstract

**Introduction:**

The cervicovaginal microbiome is a key determinant of reproductive health. Its multi-kingdom structure and ecological interactions remain insufficiently characterized across diverse populations. This study aimed to define the composition and cross-kingdom dynamics of the cervical microbiome in women without HPV infection and with normal cytology in a Kazakhstani population.

**Methods:**

In this cross-sectional study, cervical samples from 92 reproductive-age women were analyzed using whole-genome metagenomic sequencing to characterize bacterial, viral, fungal, and archaeal communities, together with predicted functional pathways. Microbial communities were stratified into community state types based on dominant bacterial species.

**Results:**

Bacterial composition differed markedly across community states, with *Lactobacillus*-dominated profiles associated with low diversity and anaerobe-rich communities associated with higher diversity. In contrast, viral, fungal, and archaeal diversity remained relatively stable, although descriptive compositional shifts indicated variation in bacteriophages, methanogenic archaea, and opportunistic fungi in non-*Lactobacillus* communities. Functional analyses indicated CST-associated pathway differences, suggesting greater metabolic flexibility in dysbiotic states, and exploratory network analysis revealed CST-associated restructuring of bacterial and cross-kingdom co-variation patterns. Notably, more than half of participants exhibited non-*Lactobacillus*-dominated communities despite the absence of infection or cytological abnormalities, indicating population-specific microbiome configurations.

**Discussion:**

Study demonstrates that the cervical microbiome is accompanied by exploratory cross-kingdom compositional variation and ecological states traditionally considered dysbiotic may represent stable, population-specific configurations, highlighting the need for context-dependent definitions of microbial health.

## 1 Introduction

The cervix represents a critical anatomical and biological interface between the lower and upper female reproductive tract, playing an essential role in reproductive health and disease susceptibility. The cervical epithelium within transformation zone is continuously exposed to endogenous hormonal fluctuations and exogenous microbial influences, thus becoming the most vulnerable part for the infections. This underscores the key role of the cervicovaginal microbiome in local mucosal homeostasis (Chao et al., 2021). In healthy reproductive-age individuals, the cervicovaginal microbiome is most commonly characterized by low microbial diversity and dominance of *Lactobacillus* species, which are associated with low pH, protective metabolic functions and protection through adherence to mucous and disruption of biofilms (Mitra et al., 2015). Dominant species in a healthy profile commonly include *L. crispatus*, *L. iners*, *L. gasseri* and *L. jensenii* (Kyrgiou and Moscicki, 2022). By producing lactic-acid and maintaining acidic environment, that suppress colonization by pathogenic and opportunistic microbiota.

In contrast, Bacterial vaginosis (BV) is associated with the disruption in the healthy microbiome and sudden replacement of *Lactobacilli* by anaerobic bacteria such as *Gardnerella vaginalis*, *Atopobium vaginae*, *Ureaplasma urealyticum*, *Mycoplasma hominis*, and others (Abou Chacra et al., 2022). This ecological variation is commonly described using community state types (CSTs), which classify cervicovaginal communities according to dominant bacterial profiles (De Seta et al., 2019). However, *Lactobacillus* dominance does not represent a uniform protective state. *L. crispatus*-dominated communities are generally considered more stable (Guan et al., 2023), whereas *L. iners* may occur both in *Lactobacillus*-dominated and more diverse transitional communities (Brooks et al., 2017; Łaniewski et al., 2020). CST stability is also influenced by host and behavioral factors, including menstrual cycle phase, hormonal variation, sexual behavior (Ybaseta-Medina et al., 2026), and cervical epithelial characteristics (Aggarwal and Ben Amor, 2025; Cambridge University Hospitals, 2026). Regularity of the menstrual cycle, phase of the cycle and number of partners are one of the crucial determinants of the microbial diversity too. According to other studies, *Lactobacillus* abundance decreased during menstruation at the genus level, however, no universal increases or decreases during menses were found at the species level (Song et al., 2020). Therefore, species-level and population-specific characterization is important for distinguishing stable *Lactobacillus*-dominated states from transitional or dysbiosis-like microbial configurations. Because cervicovaginal microbiome composition varies substantially across populations, defining local baseline microbial profiles is also essential for interpreting microbiome-associated risk in HPV-related cervical disease. This is particularly relevant in regions where cervical cancer and HPV-related disease remain major public health concerns, but where population-specific cervicovaginal microbiome data are limited.

In Kazakhstan cervical cancer remains a public health burden with persistently high incidence over the past two decades. Recent national data showing age-standardized incidence and mortality in 2023 was 16.36 and 6.61 per 100,000 women, respectively (Rakhmankulova et al., 2026). This burden is closely linked to high-risk human papillomavirus (HPV), the established causal factor in cervical carcinogenesis, with local studies reporting high-risk HPV prevalence of 39%–43% among women attending gynecological clinics and 62.4% among women with abnormal cytology (Babi et al., 2021). In this context, characterizing the cervicovaginal microbiome in HPV-negative women with normal cytology provides an important baseline for understanding population-specific microbial patterns before disease development and for interpreting future HPV-related prevention strategies in Kazakhstan. While numerous studies have examined cervical microbiome alterations in the context of HPV infection and cervical intraepithelial neoplasia, fewer investigations have focused on microbiome variation in HPV-negative individuals with normal cytology. Existing studies suggest that the composition of the normal cervicovaginal microbiome varies across populations and ethnic groups, with *Lactobacillus*-dominant communities reported more frequently in White and Asian women than in Hispanic, Black, and some African populations (Ravel et al., 2011).

Therefore, this study aimed to characterize the cervicovaginal microbiome of HPV-negative women with normal cytology in Kazakhstan, representing an understudied Central Asian population. The conceptual novelty of this study lies in moving beyond a bacteria-centered description of cervicovaginal community state types toward a multi-kingdom ecological interpretation. Rather than defining CSTs only by dominant bacterial taxa, we examined whether *Lactobacillus*-dominated, transitional, and non-*Lactobacillus*-dominated states were accompanied by parallel shifts in viral, fungal, and archaeal profiles. This approach allowed us to evaluate CSTs as broader ecological configurations reflecting bacterial dominance, phage-associated community structure, opportunistic fungal representation, and low-abundance archaeal signatures. Thus, the study provides not only a population-specific microbiome baseline for HPV-negative women with normal cytology, but also an ecological framework for understanding cervicovaginal community variation across multiple microbial kingdoms.

## 2 Materials and methods

### 2.1 Study design and population

A cross-sectional study was conducted at the tertiary care academic medical center in Astana, Kazakhstan. Participant recruitment was carried out at the National Research Center for Mother and Child Health through the outpatient gynecology clinic. Eligible participants were non-pregnant women aged 18–46 years with a regular menstrual cycle and an intact uterine cervix. Women were excluded if they had severe chronic or immunocompromising conditions, acute inflammatory disease at the time of recruitment, recent antibiotic/probiotic/immunosuppressive therapy, smoking history, intrauterine device use, prior HPV vaccination, or recent invasive gynecological procedures. The present analysis used a subset of participants from this parent cross-sectional study (Ukybassova et al., 2025). Samples with HPV positivity by PCR or WGS-based screening, abnormal cytology, incomplete metadata, insufficient sequencing data, or missing CST classification were excluded from the final analytical cohort.

### 2.2 Sample collection, DNA extraction, and metagenomic sequencing

Cervical samples were collected using sterile swabs during routine gynecological examination and stored at −80 °C until processing. Total genomic DNA was extracted using the ZymoBIOMICS DNA Miniprep Kit (Zymo Research, Irvine, CA, USA) according to the manufacturer’s protocol. Sterile nuclease-free water served as a negative extraction control, and the ZymoBIOMICS Microbial Community Standard was included as a positive control.

DNA concentration was measured using a Qubit Fluorometer, and DNA purity was assessed using the UNano-1000 spectrophotometer. Sample integrity was evaluated using the Qsep400 Analyzer. DNA samples were required to have a total amount of at least 1.0 μg, sufficient for duplicate library construction, with good integrity and no visible impurities. Whole-genome shotgun metagenomic sequencing was performed on the Illumina NovaSeq 6000 platform using a paired-end 150 bp strategy, with an expected output of 6 Gb raw data per sample. To minimize host-derived background, paired-end reads were aligned to the human GRCh38 reference genome using Bowtie2 v2.5.4 prior to microbial taxonomic classification. Read pairs failing concordant alignment to the human genome were retained with the –un-conc-gz option and considered non-host reads. For each sample, the retained forward and reverse read files were concatenated into a single FASTQ file for subsequent Kraken2/Bracken-based taxonomic profiling.

Taxonomic classification was performed using Kraken2 v2.1.3 against the PlusPFP Kraken2/Bracken prebuilt database. The PlusPFP database contains the standard Kraken2 RefSeq libraries, including archaea, bacteria, viral genomes, plasmids, human, and UniVec_Core, supplemented with RefSeq protozoa, fungi, and plant genomes. This database was selected to enable broad-spectrum taxonomic profiling of bacterial, archaeal, viral, fungal, and eukaryotic microbial reads from cervical shotgun metagenomic data. Bracken v3.0 was subsequently applied to refine abundance estimates at the species and genus levels. Functional profiling was performed with the HUMAnN 3.0 pipeline, utilizing UniRef90 for protein sequence annotation and metabolic pathway reconstruction against the MetaCyc pathway database.

To contextualize multi-kingdom read assignment, Kraken2 reports were used to summarize classified reads at the kingdom/domain level. For each sample, reads assigned to Bacteria, Viruses, Fungi, and Archaea were extracted from the corresponding Kraken2 report. Only samples included in the final analytical cohort were retained. The proportion assigned to each kingdom/domain was calculated relative to the total reads assigned to these four microbial groups and summarized as median values.

### 2.3 HPV status classification

Human papillomavirus status was assessed using a real-time PCR assay (RealBest DNA HPV High Carcinogenic Risk Genotype kit, Cat# C8899, Vector-Best), which detects high-risk HPV genotypes 16, 18, 31, 33, 35, 39, 45, 51, 52, 56, 58, and 59. Samples were classified as HPV-positive if at least one high-risk genotype was detected (Ct ≤ 35) and HPV-negative if none were identified according to the manufacturer’s criteria.

In addition, whole-genome shotgun (WGS) sequencing data were screened for HPV sequences. Because WGS enables broader genotype detection beyond the PCR panel, samples in which WGS identified any HPV type were classified as HPV-positive. All HPV-positive samples were excluded from the current analysis.

### 2.4 Microbial community state type (CST) assignment

Samples were stratified into community state types (CSTs) based on species-level relative abundance profiles. Three biologically defined CSTs were included in downstream analyses:

CST I - *Lactobacillus crispatus*-dominant
CST III - *Lactobacillus iners*-dominant
CST IV - non-*Lactobacillus*-dominant (anaerobe-rich)

Samples were assigned to CSTs using species-level bacterial relative abundance profiles. The dominant bacterial group was defined as the group with the highest cumulative relative abundance in each sample. *Lactobacillus*-dominated samples were classified according to the most abundant *Lactobacillus* species: CST I for *Lactobacillus crispatus* and CST III for *Lactobacillus iners*. Samples dominated by *Gardnerella* or other non-*Lactobacillus* taxa were classified as CST IV, representing non-*Lactobacillus*-dominant/anaerobe-rich communities.

### 2.5 Data analysis

All microbiome, functional, and network analyses were performed in Python v3.12 using NumPy (v2.0.1) (Harris et al., 2020), SciPy (v1.15.1) (Virtanen et al., 2020), Pandas (The Pandas Development Team, 2026), statsmodels (v0.14.4), scikit-learn (v1.6.1), Matplotlib (v3.10.0) (Hunter, 2007), seaborn (v0.13.2) (Waskom, 2021). Demographic and baseline clinical characteristics were analyzed using Stata v18 (StataCorp, College Station, TX, USA). Continuous variables were compared across CST groups using the Kruskal-Wallis test, and categorical variables were evaluated using Pearson’s chi-squared test, as appropriate. Relative abundance of taxonomic data was calculated using total sum scaling (TSS). Although CST I in this dataset demonstrated relatively higher median classified read counts (median number of classified reads: 211,198 for CST I, 111,406 for CST III, and 182,879 for CST IV), no increase in diversity metrics was observed for CST I; thus, no rarefaction or depth bias adjustment was performed. Taxonomic relative abundance tables were filtered to remove low-prevalence taxa prior to downstream analyses using a 50% threshold.

Because fungal reads represented a low-abundance component of the metagenomic profiles and may be vulnerable to environmental or reagent-associated contamination, an additional contamination-aware curation step was applied to the fungal dataset. Species-level fungal taxa were screened according to taxonomic family, genus, and species. Taxa with strong environmental, soil-associated, plant-associated, or otherwise low-plausibility origin for the cervicovaginal niche were excluded before downstream fungal composition analyses. The complete list of excluded taxa is provided in Supplementary Table 1. After filtering, fungal relative abundances were recalculated by renormalizing the remaining fungal taxa within each sample.

Differential taxonomic abundance analysis was performed using MaAsLin2 to identify taxa associated with CST membership. Analyses were conducted separately for bacterial, viral, fungal, and archaeal abundance profiles at the taxonomic levels used in the composition figures. CST group was included as the fixed effect, with CST I used as the reference category. MaAsLin2 was run using total-sum scaling normalization, log transformation, linear modeling, and Benjamini-Hochberg false discovery rate correction. Positive coefficients indicated enrichment relative to CST I, whereas negative coefficients indicated depletion relative to CST I. Taxa with FDR-adjusted *q*-values < 0.05 were considered statistically significant.

Alpha diversity was assessed using Observed richness, Shannon diversity, and Pielou evenness, representing taxon richness, richness-weighted diversity, and evenness of taxon distribution, respectively. Differences across CST groups were tested using the Kruskal-Wallis test. Beta diversity was assessed using Bray-Curtis dissimilarity and visualized by principal coordinates analysis (PCoA). PERMANOVA was used to test differences in overall community composition across CST groups, while PERMDISP was used to evaluate whether these differences were influenced by unequal within-group dispersion.

Functional pathway profiles generated from shotgun metagenomic data were analyzed to assess CST-associated functional differences. Pathway abundances were tested using MaAsLin2, with CST group included as the main explanatory variable and CST I used as the reference category. *P*-values were adjusted for multiple testing using the Benjamini-Hochberg false discovery rate procedure. Pathways with FDR-adjusted q < 0.05 were considered statistically significant. Functional results not meeting this threshold were interpreted as exploratory and were not used to support definitive biological conclusions.

### 2.6 Exploratory functional, machine-learning, and network analyses

As an exploratory supplementary analysis, Random Forest classification was used to evaluate whether fungal, archaeal, or viral profiles could distinguish *Lactobacillus*-dominated communities from CST IV/non-*Lactobacillus*-dominated communities. Bacterial profiles were not modeled because CST assignment was based on bacterial composition. Low-prevalence and low-abundance taxa were filtered, relative abundance data were centered log-ratio transformed with a pseudocount, and model performance was evaluated using stratified cross-validation, ROC-AUC, balanced accuracy, F1-score, confusion matrices, and permutation-based feature importance.

Exploratory bacterial species-level co-occurrence networks were constructed separately for each CST using CLR-transformed relative abundance data and Spearman’s rank correlations. Taxa were retained if detected in at least 30% of samples within the corresponding CST, and the top 100 species by mean relative abundance were selected for visualization. Correlation *p*-values were adjusted using the Benjamini-Hochberg method, and edges were retained at | r| ≥ 0.6 and FDR-adjusted q ≤ 0.05. Network topology metrics, including number of nodes, edges, mean degree, density, and positive-to-negative edge ratio, were calculated descriptively. Exploratory cross-kingdom co-variation networks were constructed separately for each CST using bacterial species-level profiles and genus-level profiles for fungi, archaea, and viruses. Only cross-kingdom taxon pairs were tested. Taxa were filtered by prevalence and mean abundance, CLR-transformed with a pseudocount, and assessed using Spearman correlations with Benjamini-Hochberg FDR correction. Associations were retained at | r| ≥ 0.6 and FDR-adjusted q ≤ 0.05. Both bacterial and cross-kingdom networks were interpreted as exploratory co-variation patterns rather than evidence of direct ecological interactions, particularly given the modest sample sizes in CST I and CST III. All statistical analyses were interpreted with attention to both statistical significance and effect size. For analyses involving multiple comparisons, *p*-values were adjusted using the Benjamini-Hochberg false discovery rate procedure. Associations were considered statistically significant only when they met the predefined significance criteria, including FDR-adjusted *q*-values below the specified threshold. For correlation-based co-occurrence and cross-kingdom analyses, both the direction and magnitude of association were reported using Spearman correlation coefficients, and only associations with | r | ≥ 0.6 and FDR q ≤ 0.05 were interpreted as significant. Non-significant results were reported as such and were not interpreted as evidence of biological differences.

### 2.7 Availability of data and materials

Raw metagenomic sequencing data have been deposited in NCBI under BioProject accession number PRJNA1337009. Analysis code and reproducible workflows are available upon reasonable request.

## 3 Results

### 3.1 Demographic and clinical data

A total of 92 women were included and stratified into CST I (*L. crispatus*, *n* = 19), CST III (*L. iners*, *n* = 22), and CST IV (non-*Lactobacillus*-dominated, *n* = 51) (Supplementary Table 2). The median age was 34.2 years (IQR 31.4–39.6) and did not differ across CSTs (*p* = 0.71). Most participants were Kazakh (92%). BMI, cycle characteristics, number of sexual partners, education, marital status, parity, and most clinical variables were comparable across CSTs. Hormonal contraception differed significantly across CST groups (*p* = 0.011), with the highest frequency in CST III, while allergy prevalence was also different across CSTs (*p* = 0.037). In multivariable multinomial logistic regression using CST I (*L. crispatus*-dominant) as the reference category, menstrual cycle phase was not significantly associated with CST III or CST IV membership. Later age at menarche was associated with higher relative risk of CST III membership (RRR = 2.68, 95% CI: 1.24–5.82, *p* = 0.013) and CST IV membership (RRR = 2.12, 95% CI: 1.06–4.21, *p* = 0.033) compared with CST I. Contraception use showed borderline but non-significant associations with CST III (RRR = 4.91, 95% CI: 0.93–25.95, *p* = 0.061) and CST IV (RRR = 4.23, 95% CI: 0.95–18.86, *p* = 0.059).

In our study, median *Lactobacillus* relative abundance was significantly lower during menses compared to non-menstrual phases (2.64 [IQR 0.22–2.90] vs. 22.00 [IQR 1.02–81.14], Wilcoxon *p* = 0.041) (Supplementary Table 3). In contrast, *Gardnerella* abundance did not differ significantly between phases (*p* = 0.92).

### 3.2 Relative abundance of bacterial, archaeal, viral and fungal domains

Most classified microbial reads were bacterial, with a median bacterial read 118,233.0 (99.844%). Fungal, viral, and archaeal reads represented substantially smaller fractions, with median counts of 129 (0.105%), 26 (0.023%), and 5 (0.005%) reads per sample, respectively.

Combined composition figures were used to summarize both cohort-level mean relative abundance and individual-level microbiome profiles across CSTs for bacteria, fungi, archaea, and viruses. Bacterial and viral compositions are presented at species level, whereas fungal and archaeal profiles are shown at genus level because species-level assignments were less reliable and included low-confidence taxa, likely due to the lower abundance and limited taxonomic resolution of these kingdoms in cervical metagenomic data. The species level for them are presented in the Supplementary Figures 1, 2.

#### 3.2.1 Bacterial community composition

**FIGURE 1:**
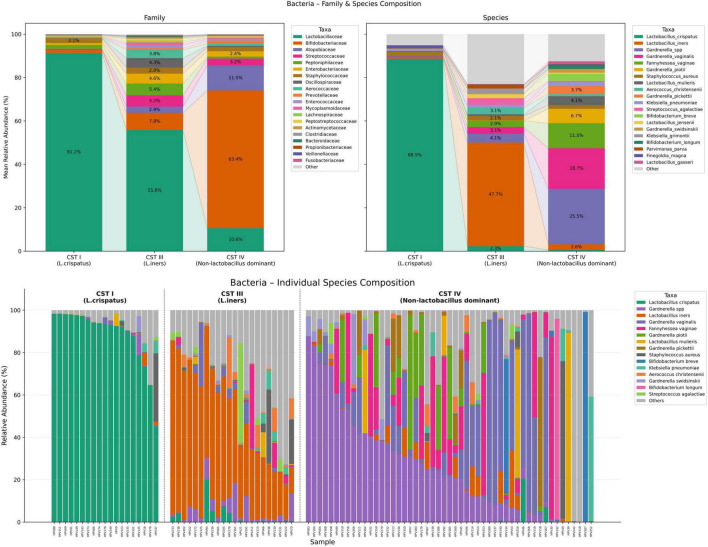
Bacterial family- and species-level composition across cervicovaginal community state types in HPV negative, NILM women.

At the family level, CST I was strongly dominated by Lactobacillaceae, which accounted for 91.2% of mean relative abundance (Figure 1). This dominance was partially retained in CST III, where Lactobacillaceae decreased to 55.8%, together with increased representation of Bifidobacteriaceae, Streptococcaceae, Peptoniphilaceae, Enterobacteriaceae, and other lower-abundance families. CST IV showed the most pronounced restructuring, with Bifidobacteriaceae becoming the dominant family at 63.4%, while Lactobacillaceae decreased sharply to 10.6%. Atopobiaceae also increased to 11.5%, supporting a shift toward a more diverse, non-*Lactobacillus*-dominated anaerobic community.

At the species level, CST I was characterized by near-monodominance of *Lactobacillus crispatus* (88.5%), indicating a relatively stable and low-diversity bacterial state. CST III was primarily driven by *Lactobacillus iners* (47.7%), while *L. crispatus* declined to 2.3%, consistent with a less stable *Lactobacillus*-dominated state. CST IV differed substantially from both *Lactobacillus*-dominated CSTs and was characterized by expansion of *Gardnerella* spp. (25.5%), *Gardnerella vaginalis* (18.7%), *Fannyhessea vaginae* (11.5%), and *Gardnerella piotii* (6.7%), while *L. iners* contributed only 2.6%. The individual-level profiles reinforced this pattern: CST I showed relatively homogeneous *L. crispatus* dominance, CST III showed more variable *L. iners* dominance, and CST IV showed pronounced inter-individual heterogeneity with alternating dominance of *Gardnerella*, *Fannyhessea*, and other anaerobic taxa.

#### 3.2.2 Differential taxonomic abundance analysis

To formally evaluate CST-associated taxonomic differences, MaAsLin2 differential abundance analysis was performed using CST I as the reference group (Supplementary Table 4). Significant associations after FDR correction were observed only in the bacterial fraction (Supplementary Figure 3). At the species level, CST III was characterized by enrichment of *Lactobacillus iners* and depletion of *Lactobacillus crispatus* relative to CST I. CST IV showed enrichment of *Gardnerella*-associated taxa, including *Gardnerella vaginalis*, *Gardnerella piotii*, and multiple *Gardnerella* spp., together with depletion of *L. crispatus* and other *Lactobacillus* species. No fungal, archaeal, or viral taxa remained significant after FDR correction at q < 0.05. These findings support bacterial taxa as the primary CST-defining signal, whereas non-bacterial kingdom-level patterns should be interpreted as exploratory.

#### 3.2.3 Viral community composition

**FIGURE 2:**
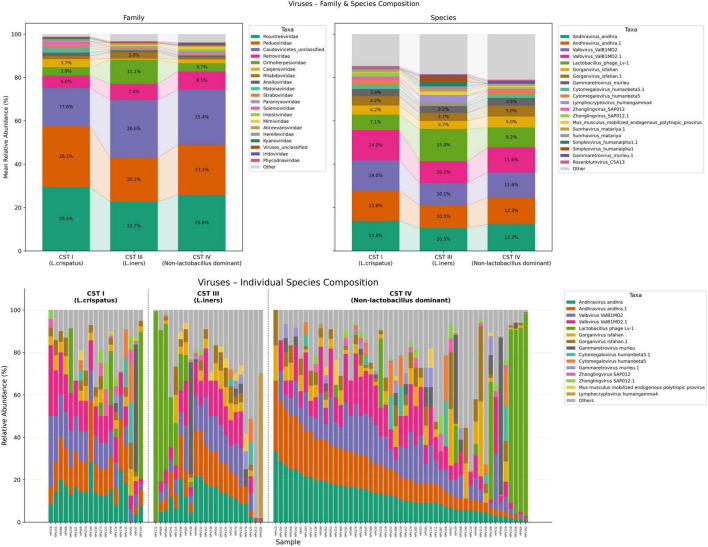
Viral family- and species-level composition across cervicovaginal community state types in HPV negative, NILM women.

The viral community was largely composed of bacteriophage-associated taxa, suggesting that the virome may partly reflect the underlying bacterial community structure (Figure 2). At the family level, CST I was dominated by Rountreeviridae (29.5%) and Peduoviridae (28.1%), followed by unclassified Caudoviricetes (17.6%). In CST III, Rountreeviridae and Peduoviridae decreased to 22.7% and 20.2%, respectively, whereas unclassified Caudoviricetes increased to 26.6%. CST IV showed a similar phage-rich profile, with Rountreeviridae (25.8%), unclassified Caudoviricetes (25.4%), and Peduoviridae (23.1%) contributing comparable proportions. Retroviridae increased modestly from CST I (5.6%) to CST III (7.4%) and CST IV (8.5%), although it did not dominate any CST.

At the species level, CST I contained comparable contributions from *Andhravirus* and *Valbvirus* lineages, each contributing approximately 13.8%–14.0%, together with *Lactobacillus* phage Lv-1 at 7.1%. In CST III, *Lactobacillus* phage Lv-1 increased to 15.0%, while *Andhravirus* and *Valbvirus* lineages decreased to approximately 10.1%–10.5%. In CST IV, *Andhravirus* and *Valbvirus* lineages remained among the most abundant viral taxa, each contributing approximately 11.6%–12.3%, while *Lactobacillus* phage Lv-1 decreased to 9.2%. Overall, the viral profiles suggest CST-associated variation in phage composition, but the individual-level plots indicate substantial within-CST heterogeneity, implying that viral structure was less tightly coupled to CST classification than bacterial composition.

#### 3.2.4 Archaeal community composition

**FIGURE 3:**
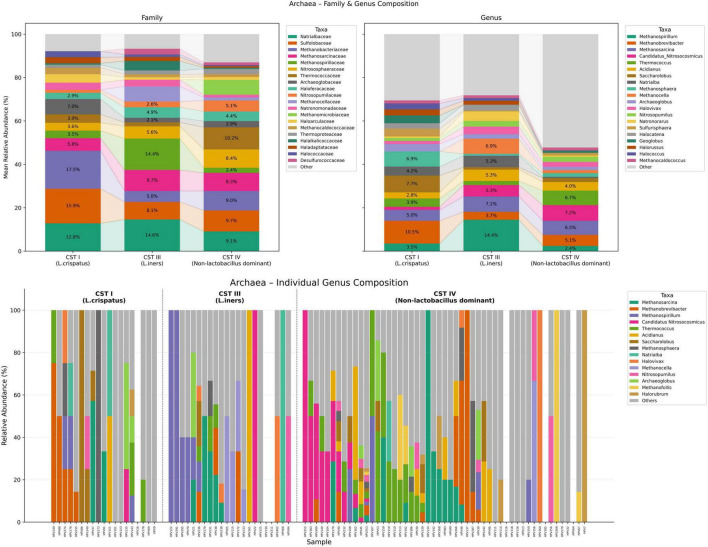
Archaeal family- and genus-level composition across cervicovaginal community state types in HPV negative, NILM women.

Given the very low archaeal read fraction, archaeal composition was interpreted descriptively. Archaeal profiles were more heterogeneous than bacterial profiles and contained a larger proportion of low-abundance or unclassified taxa, especially at the genus level (Figure 3). At the family level, CST I included relatively higher proportions of Methanobacteriaceae (17.5%), Sulfolobaceae (15.9%), and Natrialbaceae (12.8%). In CST III, Natrialbaceae increased to 14.6%, Methanospirillaceae to 14.4%, and Methanosarcinaceae to 9.7%, while Methanobacteriaceae decreased to 5.0%. CST IV showed a more evenly distributed archaeal profile, including Thermococcaceae (10.2%), Sulfolobaceae (9.7%), Natrialbaceae (9.1%), Methanobacteriaceae (9.0%), Methanosarcinaceae (8.3%), and Nitrososphaeraceae (8.4%).

At the genus level, CST I showed contributions from *Methanobrevibacter*, *Acidianus*, *Natrialba*, and *Methanospirillum*, whereas CST III showed higher *Methanosarcina* (14.4%) together with *Methanospirillum* and *Natrialba*. CST IV had no single dominant archaeal genus and showed a large contribution from “Other” taxa, suggesting a more even distribution of detected archaeal taxa and weaker CST-specific structure. Therefore, unlike bacteria, archaeal composition should be interpreted as a heterogeneous background signal rather than a primary driver of CST separation.

#### 3.2.5 Fungal community composition

**FIGURE 4:**
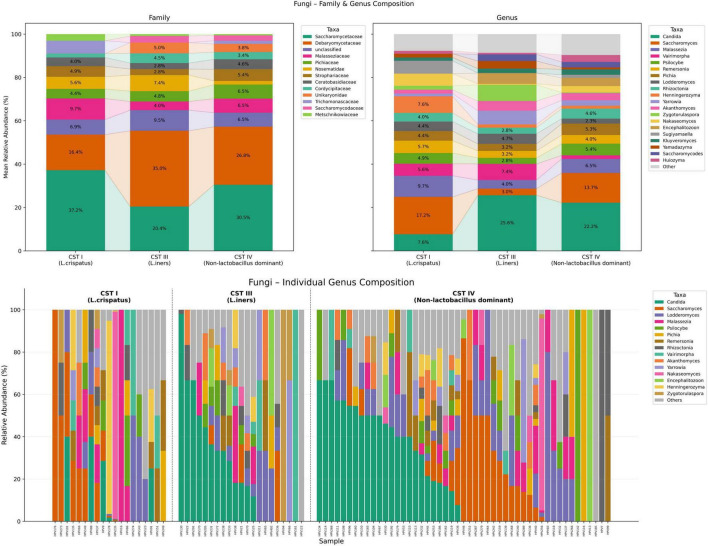
Fungal family- and genus-level composition across cervicovaginal community state types in HPV negative, NILM women.

Fungal composition also varied across CSTs, but the pattern was less uniform than in bacteria. At the family level, CST I was enriched in Saccharomycetaceae (37.2%), followed by Debaryomycetaceae (16.4%), Malasseziaceae (9.7%), unclassified fungi (6.9%), and Nectriaceae (5.6%). In CST III, the profile shifted toward Debaryomycetaceae, which increased to 35.0%, while Saccharomycetaceae decreased to 20.4% (Figure 4). CST IV showed an intermediate but still altered fungal profile, with Saccharomycetaceae at 30.5% and Debaryomycetaceae at 26.8%, together with Malasseziaceae, Pichiaceae, and unclassified fungi, each contributing approximately 6.5%.

At the genus level, CST I was mainly characterized by *Saccharomyces* (17.2%), *Lodderomyces* (9.7%), *Candida* (7.6%), *Malassezia* (5.6%) and *Pichia* (5.7%). In CST III, *Candida* became the most abundant genus (25.6%), whereas *Saccharomyces* decreased to 3.0%, indicating a shift away from the *Saccharomyces*-associated profile observed in CST I. CST IV also showed high *Candida* abundance (22.2%), together with increased *Saccharomyces* (13.7%) and *Lodderomyces* (6.5%). Thus, fungal profiles suggest a transition from *Saccharomyces*/*Lodderomyces*-associated composition in CST I toward greater *Candida* representation in CST III and CST IV.

### 3.3 Alpha and beta diversities across the CST groups

#### 3.3.1 Bacterial alpha- beta - diversity

**FIGURE 5:**
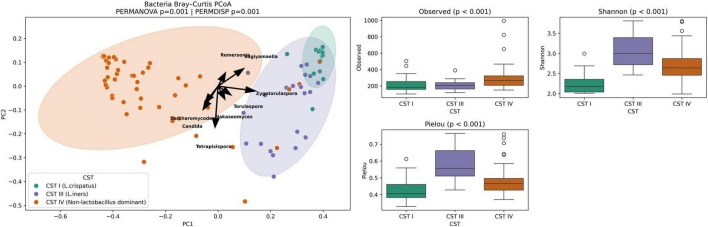
Bacterial diversity and community structure across CSTs. Bray-Curtis principal coordinate analysis (PCoA) and alpha-diversity metrics showing CST-associated differences in bacterial community composition. Beta diversity was assessed using Bray-Curtis dissimilarity, with group separation tested by PERMANOVA and dispersion assessed by PERMDISP. Alpha diversity was evaluated using Observed richness, Shannon diversity, and Pielou evenness. CST I showed the most compact clustering and lowest diversity, CST III showed the highest Shannon diversity and evenness, and CST IV demonstrated greater compositional dispersion and increased richness. Fungal vectors overlaid on the PCoA indicate taxa correlated with bacterial community gradients.

Bacterial diversity showed a clear CST-dependent gradient. CST I had the lowest richness, Shannon diversity, and evenness, consistent with a strongly *Lactobacillus crispatus*-dominated community (Figure 5). In contrast, CST IV showed the highest observed richness, while CST III had the highest Shannon diversity and Pielou evenness, indicating a more balanced distribution of bacterial taxa rather than simple dominance by one organism. All three alpha-diversity metrics differed significantly across CSTs (Observed, Shannon, and Pielou: *p* < 0.001).

Bacterial community structure also differed significantly by CST based on Bray-Curtis PCoA (PERMANOVA *p* = 0.001). CST I formed a compact cluster distinct from CST IV, whereas CST III occupied an intermediate position, supporting a compositional transition from *L. crispatus*-dominant to non-*Lactobacillus*-dominant communities. However, PERMDISP was also significant (*p* = 0.001), indicating that part of the observed beta-diversity difference was driven by unequal within-group dispersion, particularly greater heterogeneity in CST IV.

Exploratory fungal biplot vectors were overlaid on the bacterial PCoA to visualize fungal genera associated with bacterial ordination gradients. Several genera, including *Saccharomyces*, *Candida*, *Sugiyamaella*, *Zygotorulaspora*, and *Tetrapisispora*, aligned with different regions of the bacterial ordination space, suggesting descriptive co-variation between fungal profiles and CST-related bacterial restructuring.

#### 3.3.2 Alpha and beta diversity of archaeal, viral, and fungal communities

For archaeal, viral, and fungal communities, no significant CST differences were observed in richness (Observed *p* = 0.126, *p* = 0.172, *p* = 0.635), Shannon diversity (*p* = 0.119, *p* = 0.884, *p* = 0.894), or Pielou evenness (*p* = 0.277, *p* = 0.254, *p* = 0.363) (Supplementary Figure 4). Archaeal beta-diversity showed a non-significant trend toward CST separation (PERMANOVA *p* = 0.096; PERMDISP *p* = 0.448) as well as viral communities demonstrated no between-group differences (PERMANOVA *p* = 0.671; PERMDISP *p* = 0.269). Fungal beta-diversity approached significance (PERMANOVA *p* = 0.051; PERMDISP *p* = 0.210), without dispersion differences.

Exploratory pairwise taxon-level comparisons identified selected non-bacterial taxa with CST-associated patterns, but these did not correspond to broad kingdom-level diversity differences and are presented in Supplementary Figure 5.

To further assess whether non-bacterial profiles reflected bacterial CST structure, we performed exploratory Random Forest classification using fungal, viral, and archaeal profiles separately. Bacterial profiles were not modeled because CST assignment was based on bacterial community composition. Overall, non-bacterial profiles showed limited ability to distinguish *Lactobacillus*-dominated communities from CST IV, suggesting that CST structure was primarily driven by bacterial rather than fungal, viral, or archaeal variation (Supplementary Figure 6 and Supplementary Table 5).

### 3.4 Species co-occurrence networks

**FIGURE 6:**
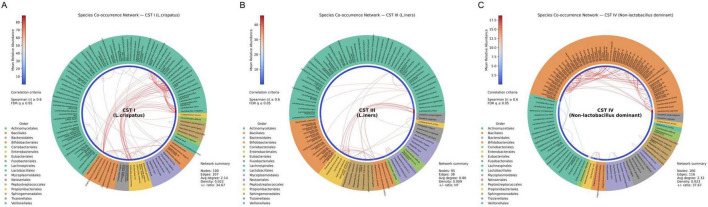
Species-level bacterial co-occurrence networks across community state types. Co-occurrence networks were constructed separately for each CST using Spearman’s rank correlation on centered log-ratio-transformed species-level relative abundance data. Taxa were retained if detected in at least 30% of samples within the corresponding CST group, and the top 100 species by mean relative abundance were included. Edges represent significant moderate-to-strong correlations after multiple-testing correction, defined as | r| ≥ 0.6 and Benjamini-Hochberg FDR-adjusted q ≤ 0.05. Red edges indicate positive correlations, and blue edges indicate negative correlations. Outer ring colors indicate bacterial order, and the inner abundance ring represents mean relative abundance. Network metrics are shown for each CST. **(A)** CST I, *Lactobacillus crispatus*-dominant community. **(B)** CST III, *Lactobacillus iners*-dominant community. **(C)** CST IV, non-*Lactobacillus*-dominant community.

Exploratory bacterial species-level co-occurrence networks were generated for each CST using CLR-transformed relative abundance data and FDR-corrected Spearman correlations (Figure 6). Edges were retained at | r| ≥ 0.6 and FDR-adjusted q ≤ 0.05. CST I included 100 nodes and 107 edges, with a mean degree of 2.14 and density of 0.022. CST III included 95 nodes and 38 edges, with a mean degree of 0.80 and density of 0.009. CST IV included 100 nodes and 116 edges, with a mean degree of 2.32 and density of 0.023. Most retained associations were positive across CSTs. These metrics suggest descriptive differences in retained bacterial co-occurrence patterns across CSTs; however, because CST I and CST III had limited sample sizes, differences in edge number, density, and mean degree may partly reflect statistical power and should not be interpreted as definitive evidence of reduced or increased ecological connectivity.

### 3.5 Cross-kingdom association patterns across CSTs

**FIGURE 7:**
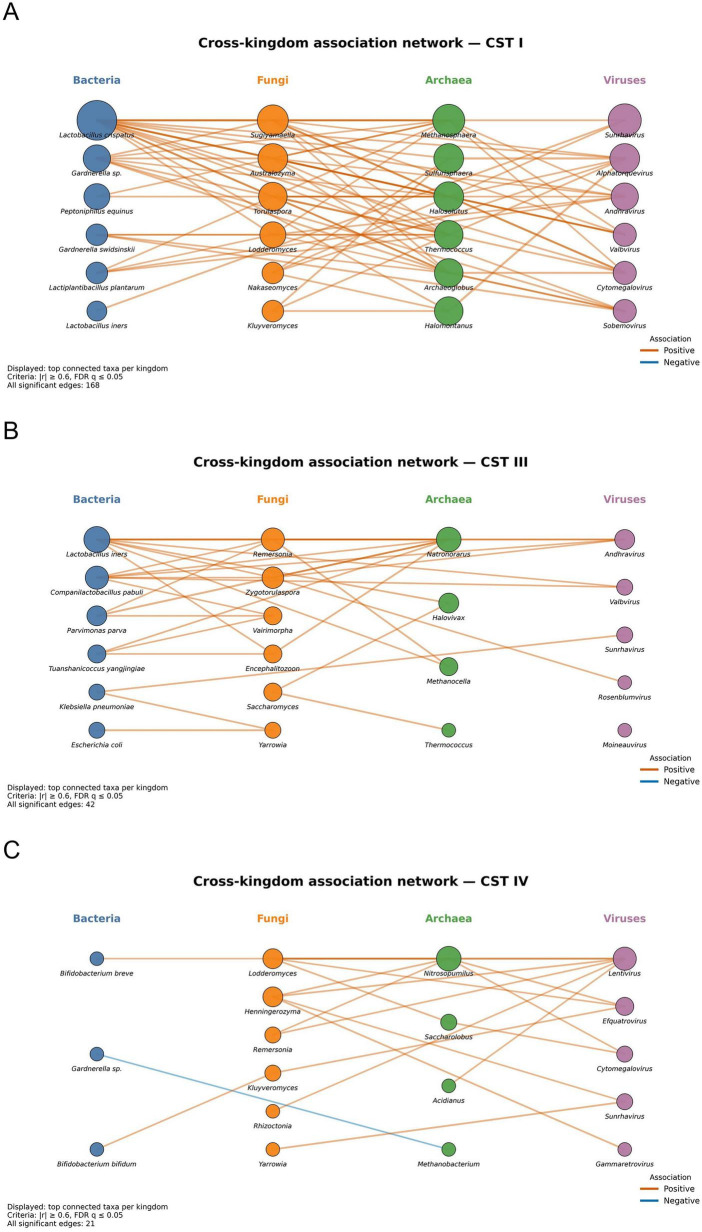
Exploratory cross-kingdom co-variation networks across CSTs. Cross-kingdom association networks were constructed separately for **(A)** CST I, dominated by *Lactobacillus crispatus*; **(B)** CST III, dominated by *Lactobacillus iners*; and **(C)** CST IV, characterized by non-*Lactobacillus*-dominated communities. Bacterial profiles were analyzed at the species level, whereas fungal, archaeal, and viral profiles were analyzed at the genus level. Taxa were filtered by prevalence and mean abundance, transformed using a centered log-ratio transformation with a pseudocount, and tested using pairwise Spearman correlations across kingdoms only. Edges indicate significant associations after Benjamini-Hochberg FDR correction (| r| ≥ 0.60, q ≤ 0.05), with orange representing positive and blue representing negative associations. For visualization, only the most connected taxa within each kingdom are displayed; therefore, the networks should be interpreted as exploratory co-variation patterns rather than direct ecological interactions.

Cross-kingdom association analysis showed CST-specific differences in association density and structure. CST I showed the highest number of significant cross-kingdom associations, with 168 edges, followed by CST III with 42 edges and CST IV with 21 edges (Supplementary Figure 7). Across all CSTs, most associations were positive, while negative associations were rare, indicating that the detected patterns mainly reflected co-occurrence (Figure 7 and Supplementary Table 6).

In CST I, associations were broadly distributed across kingdom pairs, with particularly high connectivity involving archaea-bacteria, archaea-fungi, and archaea-viruses. The network included multiple bacterial taxa, such as *Lactobacillus crispatus*, *Gardnerella* sp., *Peptoniphilus equinus*, and *Lactobacillus iners*, together with fungal genera including *Sugiyamaella*, *Torulaspora*, *Lodderomyces*, and *Kluyveromyces*. Several archaeal genera, including *Methanosphaera*, *Sulfurisphaera*, *Thermococcus*, and *Archaeoglobus*, were also highly connected. This suggests that the *L. crispatus*-dominant state had the most extensive multi-kingdom co-occurrence structure.

Community state types III showed a more limited but still detectable cross-kingdom network. Associations were mainly concentrated between bacteria and fungi, with additional archaea-linked associations. Key bacterial taxa included *Lactobacillus iners*, *Parvimonas parva*, *Klebsiella pneumoniae*, and *Escherichia coli*, while fungal genera such as *Remersonia*, *Zygotorulaspora*, *Saccharomyces*, and *Yarrowia* contributed to the network. Compared with CST I, CST III showed fewer connected taxa and a less dense association pattern.

Community state types IV had the lowest overall connectivity and a distinct association profile. Bacterial involvement was limited, mainly including *Bifidobacterium breve*, *Gardnerella* sp., and *Bifidobacterium bifidum*. In contrast, the remaining associations involved fungal genera such as *Lodderomyces*, *Kluyveromyces*, and *Yarrowia*, archaeal genera such as *Nitrosopumilus* and *Methanobacterium*, and viral genera including *Lentivirus*, *Cytomegalovirus*, and *Sunrhavirus*. This pattern suggests reduced bacterial participation in cross-kingdom associations in the non-*Lactobacillus*-dominant CST IV.

Overall, cross-kingdom association density decreased from CST I to CST III and CST IV.

### 3.6 Functional pathway comparison across CSTs

**FIGURE 8:**
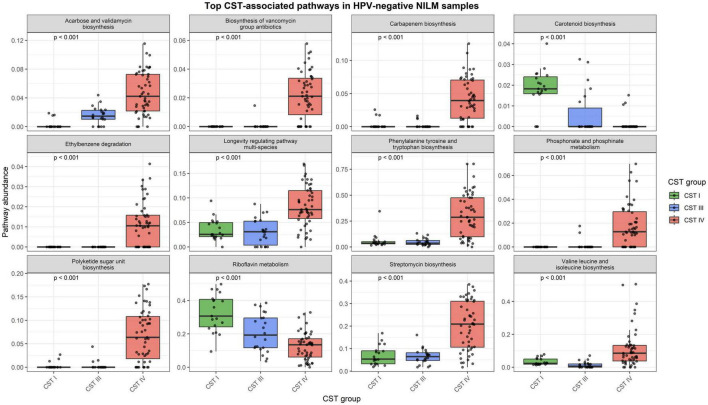
Top CST-associated functional pathways in HPV-negative NILM samples. Boxplots show the relative abundance of selected functional pathways across CST I, CST III, and CST IV. Pathways were selected from significant MaAsLin2 associations after Benjamini-Hochberg FDR correction. CST I was used as the reference group. Points represent individual samples, boxes show the interquartile range, and horizontal lines indicate medians.

Functional pathway profiling identified CST-associated differences in predicted metabolic potential among HPV-negative NILM samples (Figure 8). In the MaAsLin2 analysis using CST I as the reference group, 64 CST-associated pathway features remained significant after Benjamini-Hochberg FDR correction at q < 0.05 (Supplementary Table 7). Most significant associations were observed for CST IV compared with CST I, indicating that the non-*Lactobacillus*-dominant community state had the most distinct functional profile (Supplementary Figures 8, 9).

Community state types IV was positively associated with multiple biosynthetic and microbial metabolic pathways, including acarbose/validamycin biosynthesis, polyketide sugar unit biosynthesis, phosphonate and phosphinate metabolism, aromatic amino acid biosynthesis, phenylalanine metabolism, and sulfur metabolism. Negative associations included carotenoid biosynthesis, riboflavin metabolism, dioxin/xylene degradation, and lysine degradation (Figure 8).

Community state types III showed fewer significant functional associations than CST IV. Compared with CST I, CST III was positively associated with flagellar assembly, bacterial chemotaxis, styrene degradation, acarbose and validamycin biosynthesis, and phenylalanine metabolism. Negative associations in CST III included carotenoid biosynthesis, monobactam biosynthesis, ascorbate and aldarate metabolism, sphingolipid metabolism, dioxin degradation, xylene degradation, bile acid biosynthesis pathways, and lysine biosynthesis.

Overall, these findings indicate that CST-associated functional variation was most pronounced in CST IV, with enrichment of multiple biosynthetic, amino acid-related, and microbial metabolism pathways. CST III showed an intermediate functional profile, whereas CST I served as the reference *Lactobacillus crispatus*-dominant state.

## 4 Discussion

This study characterized the multi-kingdom cervical microbiome of HPV-negative women with normal cytology from Kazakhstan, an understudied Central Asian population. Despite the absence of HPV infection or cytological abnormalities, more than half of participants were classified as CST IV, representing non-*Lactobacillus*-dominated, anaerobe-rich communities. In contrast, CST I was characterized by strong *Lactobacillus crispatus* dominance and low bacterial diversity, whereas CST III represented an intermediate *Lactobacillus iners*-dominated state with greater compositional variability. These findings suggest that the cervical microbiome in this cohort cannot be interpreted using a single universal definition of microbial “health,” but instead requires population-specific interpretation.

### 4.1 Population-specific CST distribution

Previous studies have shown that demographic and behavioral factors, including ethnicity, sexual activity and diet, influence vaginal microbiome composition (Zeber-Lubecka et al., 2022). Regional evidence from Kazakhstan showing distinct high-risk HPV variants further supports population-specific cervicovaginal ecological patterns (Balmagambetova et al., 2024). The ethnic homogeneity of the cohort, 92% are Kazakhs, while limiting generalizability provides a relatively unconfounded Central Asian baseline that can serve as a reference for future HPV-positive and CIN-affected comparisons. Importantly, all included women were HPV-negative and had normal cytology, providing a baseline reference population rather than a disease-enriched cohort. The cohort showed a relatively low mean *Lactobacillus* abundance of 20.58%. This finding contrasts with several previously described HPV-negative or healthy cohorts in which *Lactobacillus*-dominated communities were more frequent (Ma et al., 2023). For example, a study of HPV-negative NILM women in China reported a mean *Lactobacillus* abundance of ∼60% (Ma et al., 2023; Ravel et al., 2011), whereas studies in the United States reported higher *Lactobacillus* abundance, averaging 80.2% in Asian American women and 89.7% in White women, but lower levels in Hispanic (59.6%) and Black women (61.9%) (Ravel et al., 2011). Another study also showed that 63% of healthy black women in South Africa had low *Lactobacillus* abundancy (Ravel et al., 2011). Recent studies from North Africa further support population-specific interpretation of cervicovaginal microbiome profiles. In Moroccan women, it was reported that *Lactobacillus*-dominated communities decreased across HPV-related lesion stages (Allali et al., 2025). These findings suggest that non-Western populations may show cervicovaginal microbial configurations that differ from European or North American reference profiles. Consistently, other studies have shown that alpha diversity and CST distribution vary significantly across ethnic groups, indicating that the degree of *Lactobacillus* dominance differs between populations (Wei et al., 2024). In our cohort, CST IV comprised 55% of participants, suggesting that Central Asian populations may resemble Chinese, African or Hispanic cohorts more than Western European populations in CST distribution.

Ethnic differences in cervicovaginal microbiome composition may partly reflect dietary modulation of the estrogen-mediated gut - vaginal axis (Wen et al., 2025). Gut bacteria possessing β-glucuronidase activity, including *Bacteroides* and *Clostridium* species, can influence systemic estrogen recirculation, thereby affecting vaginal epithelial glycogen availability and *Lactobacillus* colonization (Baker et al., 2017; Devkota et al., 2012). Dietary patterns may therefore indirectly shape cervicovaginal microbial composition, although these effects likely interact with host genetic, hormonal and immunological factors.

The role of the menstrual cycle in driving CST distribution deserves careful interpretation too. *Lactobacillus* dominance is generally highest during the follicular phase, when estrogen-driven glycogen deposition supports *Lactobacillus* colonization, and modestly declines in the luteal phase, sometimes with transient anaerobic blooms (Song et al., 2020, Du et al., 2023). In the present cohort, median *Lactobacillus* relative abundance was significantly lower during menses than in non-menstrual phases (*p* = 0.041), consistent with this physiology. However, *Gardnerella* abundance did not differ significantly by cycle phase (*p* = 0.92), and multivariable analysis did not identify menstrual phase as a significant predictor of CST III or CST IV membership. The only statistically significant demographic predictor was later age at menarche, which was independently associated with both CST III (RRR = 2.68, *p* = 0.013) and CST IV membership (RRR = 2.12, *p* = 0.033), suggesting that early reproductive maturation may be a developmental determinant of colonization ecology, though the biological mechanism requires further investigation. Together, these findings indicate that the high CST IV prevalence in this cohort is not a sampling artifact driven by cycle phase composition, but reflects a stable population-level tendency toward non-*Lactobacillus*-dominated ecology.

### 4.2 Bacterial CST structure

The bacterial composition data confirm the expected CST-defining patterns: CST I was characterized by near-monodominance of *L. crispatus* (88.5%), CST III by *L. iners* dominance (47.7%) with greater compositional variability, and CST IV by pronounced inter-individual heterogeneity centered on *Gardnerella* spp. (25.5%), *G. vaginalis* (18.7%), and *Fannyhessea vaginae* (11.5%). These patterns are consistent with prior multi-ethnic microbiome studies and classical CST framework (Brooks et al., 2017; De Seta et al., 2019). However, it is important to note that CST III in this cohort showed the highest Shannon diversity and Pielou evenness - higher even than CST IV - rather than simply being intermediate. This is unusual, as most studies position CST IV as the most diverse state (Mitra et al., 2015). The finding likely reflects the relatively balanced multi-taxon composition of CST III in this population, where *L. iners* dominance coexists with a wide and even spread of lower-abundance anaerobes, rather than a single alternative dominant taxon. This reinforces the characterization of CST III as a metabolically flexible transitional state (Mitra et al., 2020).

### 4.3 Cross-kingdom ecology: bacteriophages, archaea, and fungi as contextual participants

The multi-kingdom dimension of this study is its primary conceptual contribution, and it requires careful framing. The virome, archaeome, and mycobiome each showed descriptive CST-associated compositional shifts, but none met statistical significance thresholds for alpha or beta diversity, and none were identified by MaAsLin2 as CST-defining taxa. Cross-kingdom patterns should therefore be interpreted as exploratory ecological context rather than validated microbiome markers.

With this caveat, the compositional patterns are biologically informative. The virome was dominated by bacteriophage-associated families across all CSTs - principally Rountreeviridae, Peduoviridae, and unclassified Caudoviricetes - with *Lactobacillus* phage Lv-1 increasing in relative abundance in CST III. The persistence of bacteriophages across CST I, including a *Lactobacillus*-dominated state, indicates that phage activity is not restricted to high-diversity dysbiotic communities (Naureen et al., 2020). Phage predation may contribute to density-dependent regulation of dominant bacterial taxa, and their relative enrichment in CST III is consistent with greater bacterial host diversity and metabolic turnover driving intensified phage activity (Li et al., 2025; Naureen et al., 2020). This is analogous to patterns described in the gut virome, where phage-bacterial dynamics track host community structure (Castledine and Buckling, 2024).

The archaeal data present a more complex picture. CST I contained relatively higher proportions of Methanobacteriaceae and Sulfolobaceae, while CST III showed enrichment of Methanospirillaceae and Methanosarcinaceae - genera associated with hydrogenotrophic methanogenesis. CST IV showed a more even, less structured archaeal profile. The hypothesized role of methanogens in these communities is metabolic syntrophy: by scavenging hydrogen produced during anaerobic fermentation, methanogens can reduce thermodynamic inhibition of obligate anaerobes, facilitating continued bacterial fermentation and sustaining dysbiotic consortia (Guindo et al., 2020; Volmer et al., 2023). This role, well-established in the gut microbiome, would represent a significant but largely uncharacterized axis in cervicovaginal ecology. The presence of *Candidatus Nitrosocosmicus* and other ammonia-oxidizing archaea in CST IV further suggests that non-methanogenic archaeal taxa may have nitrogen-cycling potential. This interpretation is supported by evidence that ammonia-oxidizing archaea, including human-associated *Nitrosocosmicus* species detected on skin, can participate in ammonia oxidation; however, their functional role in the cervicovaginal niche remains speculative without metagenomic functional or metabolomic validation (Mahnert et al., 2026).

The fungal data showed a transition from *Saccharomyces/Lodderomyces*-associated composition in CST I toward greater *Candida* representation in CST III (25.6%) and CST IV (22.2%). This pattern is consistent with reduced colonization resistance in non-*Lactobacillus* communities. At the same time remaining lactobacilli proliferate and secrete lactic acid, hydrogen peroxide and bacteriocin-like compounds that suppress *Candida* hyphal formation and biofilm development (Zangl et al., 2019). This antagonistic feedback loop can lead to a transient co-dominance where *Candida* initiates the niche change and lactobacilli expand to counteract it (Zhao et al., 2023). *C. glabrata*, specifically enriched in dysbiosis states does not produce lactic acid, and its proliferation typically reflects elevated pH and ecological disturbance rather than causing it (Katsipoulaki et al., 2024). The capacity of both *Candida* spp. and *Gardnerella vaginalis* to form biofilms in the vaginal environment raises the possibility of interkingdom biofilm communities that create protected niches facilitating microbial persistence during dysbiosis (Boahen et al., 2022; Machado et al., 2016). Whether the *Candida* increase observed here is a consequence of bacterial dysbiosis or a contributing factor, cannot be determined from cross-sectional data. *Saccharomyces* retention in the analysis was based on prior reports of vaginal *S. cerevisiae* isolation (Gaziano et al., 2020; Huang et al., 2024; Sasivimolrattana et al., 2022). Its presence most likely reflects transient colonization, probiotic exposure, or dietary carriage, and it should not be treated as a CST-defining mycobiome feature.

### 4.4 Cross-kingdom co-variation networks

The cross-kingdom co-variation networks are among the most analytically novel outputs of this study, and their structure needs careful interpretation beyond the edge counts.

First, the sheer magnitude of the decline in cross-kingdom association density - from 168 edges in CST I to 42 in CST III and just 21 in CST IV - is striking even accounting for the smaller sample sizes of CST I and CST III. While reduced statistical power in smaller groups may suppress edge detection, the directionality is unambiguous and the CST IV reduction is especially pronounced despite CST IV having the largest sample (*n* = 51), where power should be greatest. This argues that the decrease in cross-kingdom connectivity in CST IV is a real ecological signal rather than a sampling artifact.

Second, the qualitative character of the associations changes across CSTs in a way that is biologically interpretable. In CST I, cross-kingdom associations are broadly distributed and archaea-centered: *Methanosphaera*, *Sulfurisphaera*, *Thermococcus*, and *Archaeoglobus* are among the most highly connected nodes, with associations spanning bacteria (*L. crispatus*, *Gardnerella* sp., *Peptoniphilus equinus*, *L. iners*), fungi (*Sugiyamaella*, *Torulaspora*, *Lodderomyces*, *Kluyveromyces*), and viruses. This architecture suggests that archaea function as ecological hubs in the *L. crispatus*-dominated state - not merely passive metabolic participants but potentially active organizers of multi-kingdom community structure, possibly through hydrogen cycling and syntrophic interactions that couple archaeal metabolism to both bacterial fermentation and indirect fungal niche regulation. The high archaea-bacteria connectivity in CST I is particularly notable because it occurs within a low-diversity *Lactobacillus*-dominated state. This implies that ecological integration does not require high bacterial diversity - rather, it may be a feature of stable, well-organized communities regardless of diversity level.

In CST III, cross-kingdom associations contract substantially and shift in character: bacteria-fungi associations become the dominant connection type, with archaea retaining a secondary role. Key bacterial nodes are *L. iners*, *Parvimonas parva*, *Klebsiella pneumoniae*, and *Escherichia coli* - a notable shift toward taxa associated with transitional or opportunistic colonization - while fungal genera *Remersonia*, *Zygotorulaspora*, *Saccharomyces*, and *Yarrowia* contribute to the network. The emergence of *K. pneumoniae* and *E. coli* as connected bacterial nodes in CST III, absent from the CST I network, may reflect the more permissive ecological environment of the *L. iners*-dominated transitional state, where colonization resistance is partially eroded but not yet replaced by the anaerobic dominance characteristic of CST IV.

Community state types IV shows the most distinctive cross-kingdom network structure: bacterial participation is reduced to *Bifidobacterium breve*, *Gardnerella* sp., and *Bifidobacterium bifidum*, while the remaining associations involve fungi (*Lodderomyces*, *Kluyveromyces*, *Yarrowia*), archaea (*Nitrosopumilus*, *Methanobacterium*), and, notably, viruses including *Lentivirus*, *Cytomegalovirus*, and *Sunrhavirus*. The withdrawal of most bacterial taxa from cross-kingdom associations in CST IV, combined with the persistence of fungal, viral and archaeal connectivity, suggests that as the bacterial community diversifies and fragments ecologically, the cross-kingdom network is maintained primarily by non-bacterial taxa. Whether this represents a fundamentally different mode of ecological organization, or occur because of the loss of the *L. crispatus*-mediated hub structure needs further investigation. Similar patterns have been suggested in vaginal virome studies, where bacteriophage composition and viral community structure vary with bacterial community status and BV-associated dysbiosis. Therefore, the persistence of viral connectivity in CST IV may reflect phage tracking of altered bacterial host availability rather than an independent viral driver of dysbiosis (Honorato et al., 2024; Jakobsen et al., 2020).

Third, across all three CSTs the vast majority of retained associations were positive, with negative (antagonistic) associations being rare. In CST IV this was most extreme, producing the highest positive-to-negative edge ratio (37.67) of any CST - reflecting the cooperative, co-occurring character of the anaerobic consortia that dominate this community state, consistent with the known tendency of BV-associated organisms to form synergistic biofilms (Chen et al., 2021). The relative scarcity of negative associations in CST I is slightly counterintuitive given that *L. crispatus*-dominated communities are typically considered antagonistic toward anaerobes. This may reflect the sparsity of anaerobic taxa in CST I limiting opportunities for antagonistic co-occurrence detection, or that the observed cross-kingdom associations in CST I primarily reflect co-habitation rather than direct competitive interactions.

Together, these network data support the interpretation that CST transitions involve a remodeling of the entire multi-kingdom ecological architecture, not just a bacterial compositional shift. The loss of archaea-centered hub connectivity from CST I to CST IV, and the parallel fragmentation of bacterial participation in cross-kingdom associations, is consistent with ecological resilience theory: stable communities tend to exhibit modular but well-connected network structures, whereas disrupted communities show reduced inter-module connectivity and loss of keystone organizers (Coyte et al., 2015). The modest sample sizes constrain confidence in individual edge-level findings, and these networks should be treated as hypothesis-generating rather than definitive. Nonetheless, the CST-associated network architecture described here provides a testable structural framework for future longitudinal studies examining whether cross-kingdom network disruption precedes or follows bacterial CST transitions.

### 4.5 Functional pathways: metabolic reorganization accompanies community restructuring

The functional pathway analysis identified 64 CST-associated pathway features significant after FDR correction, with CST IV showing the most distinct profile relative to CST I. These results align directionally with metabolomic and multi-omics studies showing that non-*Lactobacillus*-dominated communities have broader metabolic flexibility across amino acid, carbohydrate, lipid, and host-derived substrate pathways compared with *Lactobacillus*-dominated communities (Srinivasan et al., 2015). The enrichment of flagellar assembly and chemotaxis in CST III is particularly noteworthy: motility genes are typically associated with bacterial pathogenicity and environmental adaptation, suggesting that the *L. iners*-dominated transitional state may harbor a more behaviorally active bacterial community despite its intermediate diversity. The depletion of carotenoid and riboflavin biosynthesis in both CST III and CST IV relative to CST I suggests reduced antioxidant capacity in non-*Lactobacillus* communities, which may have implications for local mucosal immunity and oxidative stress management, though this requires metabolomic validation (Ashoori and Saedisomeolia, 2014; Medina-García et al., 2025).

### 4.6 Clinical implications and therapeutic relevance

This study provides several analytical anchors for future clinical work. First, the population-specific baseline of high CST IV prevalence in the absence of pathology means that CST IV should not automatically be coded as “dysbiosis” in Kazakhstani women without further longitudinal evidence linking it to adverse outcomes (HPV acquisition, cervical disease progression). Future case-control studies comparing HPV-negative and HPV-positive women in this population will need this baseline to correctly attribute risk to microbiome state rather than confounding by population-level ecology.

Second, the bacteriophage data raise the possibility that phage-based ecological engineering could be explored as a complement to probiotic approaches (Orton and Monaco, 2025). Current probiotic evidence is promising: oral administration of *L. crispatus* M247 promoted HPV clearance and increased CST I dominance in a randomized trial (Di Pierro et al., 2025), and a multi-strain synbiotic vaginal tablet achieved CST I conversion in 90% of recipients versus 11% placebo, with concurrent reduction in *Candida* spp. (Ravel et al., 2025). The mechanistic basis for *L. crispatus* restoration likely involves multiple pathways, including exopolysaccharide-mediated immune modulation (Croatti et al., 2026) and direct antimicrobial activity.

Nowadays, metronidazole-based BV treatment tends to shift communities toward *L. iners* rather than *L. crispatus* dominance, leaving women at higher risk of BV recurrence, points to a therapeutic gap that cysteine transport inhibition may help address. *L. iners* growth is dependent on L-cysteine uptake mechanisms, and selective inhibition of these transporters suppresses *L. iners* while promoting *L. crispatus* expansion (Bloom et al., 2022). In a population where CST III prevalence is 24% and the transition to CST IV is ecologically facile, this approach may be particularly relevant.

Finally, the multi-kingdom perspective implies that interventions targeting only the bacterial component of the cervicovaginal microbiome may produce incomplete or unstable outcomes. Restoration of *L. crispatus* dominance appears to reorganize not only the bacterial community but also fungal colonization resistance and potentially phage-mediated community regulation.

Clinically, this baseline profile may be useful for future studies comparing HPV-negative and HPV-positive women, particularly because *Lactobacillus* depletion and increased microbial diversity have been associated with high-risk HPV persistence and cervical intraepithelial neoplasia (Arokiyaraj et al., 2018). Thus, characterization of population-specific cervicovaginal microbiome states may complement cytology and HPV testing in future risk-stratification strategies, although longitudinal validation is required.

## 5 Strength and limitations

A major strength of this study is the integrative multi-kingdom approach, enabling simultaneous characterization of bacterial, viral, fungal and archaeal communities together with predicted functional profiles within the same cervical samples. Study provides the first Central Asian reference baseline. The sample size within individual CST groups was modest, although comparable to similar microbiome studies. The cross-sectional design limits inference regarding temporal microbiome dynamics and causal relationships. Although our analysis enabled multi-kingdom characterization of the taxonomic structure of healthy cervical samples from Kazakhstani women, it should be strongly emphasized that viral, fungal and archaeal classification is highly susceptible to false positives due to the lack of comprehensive, high-quality reference genomes for these domains relative to bacteria. Additionally, lower sampling load of non-bacterial taxa renders it harder to provide a comprehensive characterization of these biomes. Thus, the presented data should be interpreted with due caution. Functional metagenomic analyses, including antimicrobial resistance gene profiling, virulence factor detection, and MAG recovery, were not performed in the present study and will be addressed in future work.

## 6 Conclusion

This study establishes the first multi-kingdom cervicovaginal microbiome baseline for HPV-negative women with normal cytology in Kazakhstan, a Central Asian population not previously characterized at this resolution. The predominance of CST IV (55%) in the absence of infection or cytological pathology challenges the universal applicability of *Lactobacillus*-dominance criteria to this population and aligns the Kazakhstani microbiome profile more closely with patterns reported in Chinese, Hispanic, and sub-Saharan African cohorts. Therapeutic interventions targeting CST restoration (probiotics, synbiotics, targeted antibiotic regimens) should account for the fact that CST IV in this population may not represent the same degree of dysbiotic instability as in other populations, and that *L. crispatus*-targeted approaches are likely preferable to *L. iners*-inclusive ones given BV recurrence dynamics. The multi-kingdom ecological framework analyzed points toward a more complete understanding of cervicovaginal homeostasis where bacteria, viruses, archaea, and fungi form an integrated ecological network disruption and restoration of which operate across all four kingdoms simultaneously. Longitudinal studies integrating metagenomics with host transcriptomics and metabolomics are needed to determine whether the observed multi-kingdom patterns are functionally linked to cervical disease risk in this population.

## Data Availability

The datasets presented in this study can be found in online repositories. The names of the repository/repositories and accession number(s) can be found below: https://www.ncbi.nlm.nih.gov/, PRJNA1337009.
