# Supplementary material for: Multi-kingdom cervical microbiome structure in health and dysbiosis: a cross-sectional study from Kazakhstan
Source: Front Microbiol. 2026 Jul 27;17:1836889. doi: 10.3389/fmicb.2026.1836889 (PMC13454227; doi:10.3389/fmicb.2026.1836889)
Supplement: Supplementary file 1 [file Table_1.docx]

Supplementary Material

**Supplementary Table 1.** Complete list of excluded fungal taxa

| **Family** | **Genus** | **Species** |
| --- | --- | --- |
| "Cladosporiaceae", "Trichocomaceae", "Aspergillaceae", "Pleosporaceae",  "Mycosphaerellaceae", "Nectriaceae",  "Pucciniaceae", "Glomerellaceae",  "Clavicipitaceae", "Sclerotiniaceae", "Chaetomiaceae", "Podosporaeae",  "Pyriculariaceae", "Marasmiaceae", "Ustilaginaceae", "Onygenaceae",  "Cryptococcaceae", "Sordariaceae",  "Glomeraceae", "Didymellaceae",  "Ophiocordycipitaceae", "Schizosaccharomycetaceae" | "Trichoderma",  "Fusarium",  "Podospora",  "Eremothecium",  "Vanrija",  "Candidozyma",  "Brettanomyces",  "Naumovozyma",  "Scheffersomyces",  "Henningomyces",  "Cutaneotrichosporon" | "Trichoderma_asperellum",  "Trichoderma_atroviride",  "Fusarium_oxysporum",  "Podospora_pseudocasei",  "Eremothecium_sinecaudum",  "Vanrija_pseudolonga",  "Brettanomyces_bruxellensis",  “Cutaneotrichosporon_cavernicola",  "Puccinia_striiformis_f._sp._tritici",  "Lodderomyces_beijingensis" |

**Supplementary Table 2.** Cohort demographic and clinical description by CST groups

| Level | Total | CST I (L.crispatus) | CST III (L.iners) | CST IV  (Non- LD) | p-value | Test |
| --- | --- | --- | --- | --- | --- | --- |
|  | 92 | 19 | 22 | 51 |  |  |
| Age, median (IQR) | 34.22 (31.40, 39.69) | 35.07 (32.99, 40.79) | 37.56 (32.03, 39.73) | 33.23 (30.56, 38.63) | 0.71 | KW* |
| BMI, median (IQR) | 23.00 (21.00, 25.00) | 22.00 (21.00, 26.00) | 23.50 (20.00, 27.00) | 23.00 (21.00, 25.00) | 0.72 | KW* |
| Menarchelet, median (IQR) | 13.00 (13.00, 14.00) | 13.00 (12.00, 13.00) | 14.00 (13.00, 15.00) | 13.00 (13.00, 14.00) | 0.017 | KW* |
| Cycle days, median (IQR) | 28.00 (28.00, 30.00) | 28.00 (28.00, 30.00) | 28.50 (28.00, 30.00) | 28.00 (27.00, 30.00) | 0.95 | KW* |
| Length, median (IQR) | 5.00 (4.00, 6.00) | 6.00 (4.00, 7.00) | 5.00 (5.00, 6.00) | 5.00 (4.00, 6.00) | 0.28 | KW* |
| Days since lmp, median (IQR) | 18.00 (11.00, 24.00) | 18.00 (14.00, 24.00) | 22.00 (18.00, 26.00) | 13.00 (8.00, 22.00) | 0.010 | KW* |
| Partners, median (IQR) | 1.00 (1.00, 1.00) | 1.00 (1.00, 1.00) | 1.00 (1.00, 1.00) | 1.00 (1.00, 1.00) | 0.35 | KW* |
| Partners |  |  |  |  |  |  |
| 1 | 75 (83%) | 15 (83%) | 19 (90%) | 41 (80%) | 0.58 | PCS** |
| 1+ | 15 (17%) | 3 (17%) | 2 (10%) | 10 (20%) |  |  |
| Ethnicity |  |  |  |  |  |  |
| Kazakh | 85 (92%) | 18 (95%) | 21 (95%) | 46 (90%) | 0.56 | PCS** |
| Other | 2 (2%) | 1 (5%) | 0 (0%) | 1 (2%) |  |  |
| Russian | 5 (5%) | 0 (0%) | 1 (5%) | 4 (8%) |  |  |
| Education |  |  |  |  |  |  |
| Bachelor degree | 64 (70%) | 16 (84%) | 15 (68%) | 33 (65%) | 0.58 | PCS** |
| Master's degree | 13 (14%) | 2 (11%) | 3 (14%) | 8 (16%) |  |  |
| Middle professional education | 15 (16%) | 1 (5%) | 4 (18%) | 10 (20%) |  |  |
| Family |  |  |  |  |  |  |
| Divorced. Widowed | 9 (10%) | 1 (5%) | 3 (14%) | 5 (10%) | 0.92 | PCS** |
| Married | 75 (82%) | 16 (84%) | 17 (77%) | 42 (82%) |  |  |
| Not married | 8 (9%) | 2 (11%) | 2 (9%) | 4 (8%) |  |  |
| Cycle Regularity |  |  |  |  |  |  |
| Irregular | 8 (9%) | 0 (0%) | 1 (5%) | 7 (14%) | 0.14 | PCS** |
| Regular | 84 (91%) | 19 (100%) | 21 (95%) | 44 (86%) |  |  |
| Cycle Pain |  |  |  |  |  |  |
| Painful | 1 (1%) | 0 (0%) | 1 (5%) | 0 (0%) | 0.20 | PCS** |
| Painless | 91 (99%) | 19 (100%) | 21 (95%) | 51 (100%) |  |  |
| Volume |  |  |  |  |  |  |
| Not abundant | 3 (3%) | 0 (0%) | 2 (9%) | 1 (2%) | 0.19 | PCS** |
| Abundant | 89 (97%) | 19 (100%) | 20 (91%) | 50 (98%) |  |  |
| Cycle-day category |  |  |  |  |  |  |
| Early (0-7) | 11 (12%) | 1 (5%) | 1 (5%) | 9 (18%) | 0.074 | PCS** |
| Late (15-49) | 47 (52%) | 12 (63%) | 16 (73%) | 19 (38%) |  |  |
| Long/irregular (≥50) | 10 (11%) | 2 (11%) | 3 (14%) | 5 (10%) |  |  |
| Mid (8-14) | 23 (25%) | 4 (21%) | 2 (9%) | 17 (34%) |  |  |
| Phase based on LMP |  |  |  |  |  |  |
| Delayed/prolonged (>28) | 16 (17%) | 2 (11%) | 6 (27%) | 8 (16%) | 0.066 | PCS** |
| Early follicular (6-10) | 15 (16%) | 2 (11%) | 1 (5%) | 12 (24%) |  |  |
| Late follicular/ovulatory (11-16) | 20 (22%) | 5 (26%) | 3 (14%) | 12 (24%) |  |  |
| Luteal (17-28) | 36 (39%) | 10 (53%) | 12 (55%) | 14 (27%) |  |  |
| Menstrual (0-5) | 5 (5%) | 0 (0%) | 0 (0%) | 5 (10%) |  |  |
| Delivery number |  |  |  |  |  |  |
| 0 | 18 (20%) | 4 (21%) | 4 (18%) | 10 (20%) | 0.77 | PCS** |
| 1 | 17 (19%) | 4 (21%) | 2 (9%) | 11 (22%) |  |  |
| 2 | 29 (32%) | 7 (37%) | 9 (41%) | 13 (26%) |  |  |
| 3 | 27 (30%) | 4 (21%) | 7 (32%) | 16 (32%) |  |  |
| Aborts |  |  |  |  |  |  |
| 0 | 78 (87%) | 16 (84%) | 19 (90%) | 43 (86%) | 0.83 | PCS** |
| 1 | 12 (13%) | 3 (16%) | 2 (10%) | 7 (14%) |  |  |
| Miscarriage |  |  |  |  |  |  |
| 0 | 72 (78%) | 17 (89%) | 17 (77%) | 38 (75%) | 0.40 | PCS** |
| 1+ | 20 (22%) | 2 (11%) | 5 (23%) | 13 (25%) |  |  |
| Contraception |  |  |  |  |  |  |
| No | 54 (59%) | 13 (68%) | 11 (50%) | 30 (59%) | 0.49 | PCS** |
| Yes | 38 (41%) | 6 (32%) | 11 (50%) | 21 (41%) |  |  |
| Hormonal contraception |  |  |  |  |  |  |
| No | 51 (94%) | 9 (100%) | 11 (79%) | 31 (100%) | 0.011 | PCS** |
| Yes | 3 (6%) | 0 (0%) | 3 (21%) | 0 (0%) |  |  |
| Gynaecologic diseases |  |  |  |  |  |  |
| No | 66 (72%) | 14 (74%) | 13 (59%) | 39 (76%) | 0.31 | PCS** |
| Yes | 26 (28%) | 5 (26%) | 9 (41%) | 12 (24%) |  |  |
| Erosion |  |  |  |  |  |  |
| No | 40 (43%) | 8 (42%) | 12 (55%) | 20 (39%) | 0.48 | PCS** |
| Yes | 52 (57%) | 11 (58%) | 10 (45%) | 31 (61%) |  |  |
| Manipulations |  |  |  |  |  |  |
| No | 67 (73%) | 13 (68%) | 16 (73%) | 38 (75%) | 0.88 | PCS** |
| Yes | 25 (27%) | 6 (32%) | 6 (27%) | 13 (25%) |  |  |
| Colposcopy |  |  |  |  |  |  |
| No | 58 (65%) | 10 (59%) | 13 (62%) | 35 (69%) | 0.72 | PCS** |
| Yes | 31 (35%) | 7 (41%) | 8 (38%) | 16 (31%) |  |  |
| Chronic diseases | | | | | | |
| No | 66 (73%) | 12 (63%) | 13 (62%) | 41 (82%) | 0.11 | PCS** |
| Yes | 24 (27%) | 7 (37%) | 8 (38%) | 9 (18%) |  |  |
| Surgery |  |  |  |  |  |  |
| No | 52 (57%) | 9 (47%) | 12 (57%) | 31 (61%) | 0.60 | PCS** |
| Yes | 39 (43%) | 10 (53%) | 9 (43%) | 20 (39%) |  |  |
| Heredity |  |  |  |  |  |  |
| No | 81 (88%) | 16 (84%) | 18 (82%) | 47 (92%) | 0.39 | PCS** |
| Yes | 11 (12%) | 3 (16%) | 4 (18%) | 4 (8%) |  |  |
| Allergy |  |  |  |  |  |  |
| No | 69 (75%) | 10 (53%) | 17 (77%) | 42 (82%) | 0.037 | PCS** |
| Yes | 23 (25%) | 9 (47%) | 5 (23%) | 9 (18%) |  |  |
| Depth, median (IQR) | 6652.50 (1785.00, 38477.00) | 6635.00 (1972.00, 18396.00) | 1785.00 (796.00, 3652.00) | 31489.00 (5063.00, 64602.00) | <0.001 | KW* |
| GardnerellaCont, median (IQR) | 13.43 (0.85, 67.58) | 0.21 (0.03, 0.70) | 5.55 (1.45, 11.15) | 61.39 (35.63, 81.54) | <0.001 | KW* |
| LactobacillusCont, median (IQR) | 20.58 (0.79, 75.96) | 94.70 (89.61, 98.23) | 48.59 (33.15, 72.17) | 2.32 (0.07, 13.70) | <0.001 | KW* |
| ChlamydiaCont, median (IQR) | 0.60 (0.14, 3.23) | 0.53 (0.30, 3.56) | 3.06 (1.42, 7.60) | 0.16 (0.06, 1.43) | <0.001 | KW* |
| * KW = Kruskal Wallis; ** PCS = Pearson's chi-squared | | | | | | |

**Supplementary Table 3.** Bacterial mean abundance by menstrual phase

| Factor | Non-menstrual | Menstrual | p-value | Test |
| --- | --- | --- | --- | --- |
| N | 87 | 5 |  |  |
| Gardnerella, median (IQR) | 13.70 (0.84, 66.62) | 12.11 (0.92, 74.93) | 0.92 | Wilcoxon rank-sum |
| Lactobacillus, median (IQR) | 22.00 (1.02, 81.14) | 2.64 (0.22, 2.90) | 0.041 | Wilcoxon rank-sum |

**Supplementary Table 4.** Bacterial family- and species-level taxa significantly associated with CST membership by MaAsLin2. Taxa significantly associated with CST III or CST IV relative to CST I are shown. CST I was used as the reference group. Positive coefficients indicate enrichment in the comparison CST, while negative coefficients indicate lower abundance compared with CST I. P-values were adjusted using the Benjamini - Hochberg false discovery rate method. Only taxa with FDR q < 0.05 are included.

| Family | Species | coef | stderr | pval | qval | direction |
| --- | --- | --- | --- | --- | --- | --- |
| Aerococcaceae |  | 8.547 | 1.206 | 3.1E-10 | 4.7E-09 | Enriched in CST III |
| Oscillospiraceae |  | 6.203 | 0.923 | 1.6E-09 | 1.9E-08 | Enriched in CST III |
| Bifidobacteriaceae |  | 4.430 | 0.828 | 6.8E-07 | 6.3E-06 | Enriched in CST III |
| Streptococcaceae |  | 4.416 | 0.929 | 7.6E-06 | 5.8E-05 | Enriched in CST III |
| Enterococcaceae |  | 4.802 | 1.053 | 1.6E-05 | 1.1E-04 | Enriched in CST III |
| Peptoniphilaceae |  | 5.165 | 1.211 | 5.0E-05 | 2.9E-04 | Enriched in CST III |
| Peptostreptococcaceae |  | 4.652 | 1.178 | 1.6E-04 | 7.2E-04 | Enriched in CST III |
| Mycoplasmoidaceae |  | 4.738 | 1.284 | 3.9E-04 | 1.5E-03 | Enriched in CST III |
| Neisseriaceae |  | 3.015 | 1.124 | 8.7E-03 | 2.9E-02 | Enriched in CST III |
| Leptotrichiaceae |  | 3.277 | 1.247 | 1.0E-02 | 3.1E-02 | Enriched in CST III |
| Enterobacteriaceae |  | 2.285 | 0.959 | 1.9E-02 | 4.7E-02 | Enriched in CST III |
| Bifidobacteriaceae |  | 7.425 | 0.711 | 3.9E-17 | 1.8E-15 | Enriched in CST IV |
| Lactobacillaceae |  | -6.020 | 0.773 | 1.2E-11 | 2.7E-10 | Lower in CST IV |
| Aerococcaceae |  | 4.100 | 1.035 | 1.5E-04 | 7.2E-04 | Enriched in CST IV |
| Atopobiaceae |  | 6.207 | 1.613 | 2.2E-04 | 9.4E-04 | Enriched in CST IV |
| Oscillospiraceae |  | 2.886 | 0.792 | 4.5E-04 | 1.6E-03 | Enriched in CST IV |
| Staphylococcaceae |  | -1.814 | 0.755 | 1.8E-02 | 4.7E-02 | Lower in CST IV |
| Clostridiaceae |  | -1.739 | 0.728 | 1.9E-02 | 4.7E-02 | Lower in CST IV |
| Mycoplasmoidaceae |  | 2.659 | 1.102 | 1.8E-02 | 4.7E-02 | Enriched in CST IV |
| Aerococcaceae | Aerococcus_christensenii | 9.774 | 1.170 | 8.1E-13 | 1.6E-10 | Enriched in CST III |
| Lactobacillaceae | Lactobacillus_iners | 11.003 | 1.346 | 1.9E-12 | 2.6E-10 | Enriched in CST III |
| Lactobacillaceae | Lactobacillus_crispatus | -7.862 | 1.045 | 4.1E-11 | 4.8E-09 | Lower in CST III |
| Oscillospiraceae | Mageeibacillus_indolicus_UPII9_5 | 6.341 | 1.027 | 1.9E-08 | 1.3E-06 | Enriched in CST III |
| Oscillospiraceae | Mageeibacillus_indolicus | 6.341 | 1.027 | 1.9E-08 | 1.3E-06 | Enriched in CST III |
| Lactobacillaceae | Lactobacillus_crispatus_ST1 | -2.802 | 0.480 | 8.5E-08 | 4.6E-06 | Lower in CST III |
| Lactobacillaceae | Lactobacillus_helveticus_R0052 | -3.116 | 0.589 | 8.7E-07 | 3.7E-05 | Lower in CST III |
| Lactobacillaceae | Lactobacillus_crispatus_2029 | -2.914 | 0.637 | 1.5E-05 | 3.6E-04 | Lower in CST III |
| Oscillospiraceae | Amygdalobacter_nucleatus | 5.067 | 1.250 | 1.1E-04 | 1.7E-03 | Enriched in CST III |
| Lactobacillaceae | Latilactobacillus_sakei | -2.576 | 0.642 | 1.2E-04 | 1.9E-03 | Lower in CST III |
| Lactobacillaceae | Leuconostoc_carnosum | -1.311 | 0.329 | 1.4E-04 | 2.1E-03 | Lower in CST III |
| Lactobacillaceae | Lactobacillus_kullabergensis | -2.942 | 0.746 | 1.6E-04 | 2.3E-03 | Lower in CST III |
| Bifidobacteriaceae | Gardnerella_vaginalis | 5.036 | 1.324 | 2.6E-04 | 3.2E-03 | Enriched in CST III |
| Oscillospiraceae | Fastidiosipila_sanguinis | 3.720 | 0.978 | 2.6E-04 | 3.2E-03 | Enriched in CST III |
| Lactobacillaceae | Lactobacillus_helveticus_CNRZ32 | -1.496 | 0.400 | 3.2E-04 | 3.6E-03 | Lower in CST III |
| Streptococcaceae | Streptococcus_pneumoniae | 4.365 | 1.203 | 4.8E-04 | 5.0E-03 | Enriched in CST III |
| Lactobacillaceae | Companilactobacillus_paralimentarius | -1.868 | 0.517 | 5.1E-04 | 5.1E-03 | Lower in CST III |
| Lactobacillaceae | Ligilactobacillus_ruminis | -3.287 | 0.924 | 6.0E-04 | 5.8E-03 | Lower in CST III |
| Bifidobacteriaceae | Gardnerella_sp | 3.900 | 1.099 | 6.2E-04 | 5.8E-03 | Enriched in CST III |
| Leptotrichiaceae | Sneathia_vaginalis | 4.168 | 1.173 | 6.1E-04 | 5.8E-03 | Enriched in CST III |
| Streptococcaceae | Streptococcus_pyogenes | 3.689 | 1.050 | 7.0E-04 | 6.4E-03 | Enriched in CST III |
| Lactobacillaceae | Lactobacillus_amylovorus_GRL_1112 | -2.152 | 0.617 | 7.6E-04 | 6.9E-03 | Lower in CST III |
| Mycoplasmoidaceae | Ureaplasma_parvum | 3.202 | 0.939 | 9.8E-04 | 8.5E-03 | Enriched in CST III |
| Mycoplasmoidaceae | Ureaplasma_parvum | 4.782 | 1.411 | 1.0E-03 | 8.9E-03 | Enriched in CST III |
| Enterococcaceae | Enterococcus_cecorum | 3.255 | 1.041 | 2.4E-03 | 1.9E-02 | Enriched in CST III |
| Lactobacillaceae | Lactobacillus_amylovorus_GRL1118 | -1.516 | 0.485 | 2.4E-03 | 1.9E-02 | Lower in CST III |
| Oscillospiraceae | Amygdalobacter_indicium | 4.042 | 1.323 | 3.0E-03 | 2.1E-02 | Enriched in CST III |
| Peptoniphilaceae | Anaerococcus_vaginalis | 3.396 | 1.110 | 2.9E-03 | 2.1E-02 | Enriched in CST III |
| Lactobacillaceae | Lactobacillus_helveticus_DPC_4571 | -1.616 | 0.528 | 2.9E-03 | 2.1E-02 | Lower in CST III |
| Streptococcaceae | Streptococcus_constellatus | 3.508 | 1.148 | 3.0E-03 | 2.1E-02 | Enriched in CST III |
| Streptococcaceae | Streptococcus_agalactiae | 4.109 | 1.345 | 3.0E-03 | 2.1E-02 | Enriched in CST III |
| Streptococcaceae | Streptococcus_mitis | 3.624 | 1.193 | 3.1E-03 | 2.2E-02 | Enriched in CST III |
| Enterococcaceae | Enterococcus_dispar | 1.949 | 0.644 | 3.2E-03 | 2.3E-02 | Enriched in CST III |
| Clostridiaceae | Clostridium_caseinilyticum | 2.324 | 0.772 | 3.4E-03 | 2.3E-02 | Enriched in CST III |
| Lactobacillaceae | Lactobacillus_jensenii | 4.036 | 1.343 | 3.4E-03 | 2.4E-02 | Enriched in CST III |
| Peptoniphilaceae | Peptoniphilus_genitalis | 3.620 | 1.208 | 3.5E-03 | 2.4E-02 | Enriched in CST III |
| Lactobacillaceae | Lactobacillus_ultunensis | -2.547 | 0.860 | 3.9E-03 | 2.6E-02 | Lower in CST III |
| Lactobacillaceae | Fructilactobacillus_hinvesii | 1.451 | 0.492 | 4.0E-03 | 2.7E-02 | Enriched in CST III |
| Lactobacillaceae | Lactobacillus_helveticus_H9 | -1.564 | 0.535 | 4.4E-03 | 2.9E-02 | Lower in CST III |
| Bifidobacteriaceae | Gardnerella_sp | 3.224 | 1.176 | 7.4E-03 | 4.6E-02 | Enriched in CST III |
| Enterococcaceae | Enterococcus_faecalis | 3.489 | 1.287 | 8.0E-03 | 4.9E-02 | Enriched in CST III |
| Lactobacillaceae | Lactobacillus_crispatus | -13.553 | 0.897 | 2.7E-26 | 2.2E-23 | Lower in CST IV |
| Lactobacillaceae | Lactobacillus_crispatus_2029 | -4.941 | 0.547 | 3.1E-14 | 1.3E-11 | Lower in CST IV |
| Lactobacillaceae | Lactobacillus_helveticus_R0052 | -4.243 | 0.506 | 6.8E-13 | 1.6E-10 | Lower in CST IV |
| Lactobacillaceae | Lactobacillus_crispatus_ST1 | -3.401 | 0.412 | 1.3E-12 | 2.1E-10 | Lower in CST IV |
| Lactobacillaceae | Lactobacillus_kullabergensis | -4.567 | 0.640 | 2.5E-10 | 2.5E-08 | Lower in CST IV |
| Bifidobacteriaceae | Gardnerella_vaginalis | 7.638 | 1.137 | 1.7E-09 | 1.5E-07 | Enriched in CST IV |
| Lactobacillaceae | Lactiplantibacillus_plantarum | -6.199 | 0.995 | 1.5E-08 | 1.2E-06 | Lower in CST IV |
| Lactobacillaceae | Lactobacillus_acetotolerans | -4.181 | 0.698 | 4.4E-08 | 2.8E-06 | Lower in CST IV |
| Lactobacillaceae | Lactobacillus_amylovorus_GRL_1112 | -3.124 | 0.530 | 6.5E-08 | 3.8E-06 | Lower in CST IV |
| Bifidobacteriaceae | Gardnerella_sp | 5.945 | 1.027 | 1.0E-07 | 5.3E-06 | Enriched in CST IV |
| Lactobacillaceae | Lactobacillus_helveticus | -4.050 | 0.721 | 2.2E-07 | 1.1E-05 | Lower in CST IV |
| Bifidobacteriaceae | Gardnerella_piotii | 6.774 | 1.245 | 4.6E-07 | 2.1E-05 | Enriched in CST IV |
| Bifidobacteriaceae | Gardnerella_sp | 6.184 | 1.178 | 1.0E-06 | 3.7E-05 | Enriched in CST IV |
| Lactobacillaceae | Lactobacillus_ultunensis | -3.873 | 0.738 | 1.0E-06 | 3.7E-05 | Lower in CST IV |
| Lactobacillaceae | Lactobacillus_amylovorus | -4.560 | 0.869 | 1.0E-06 | 3.7E-05 | Lower in CST IV |
| Lactobacillaceae | Lactobacillus_amylovorus_DSM_20531 | -3.790 | 0.722 | 1.0E-06 | 3.7E-05 | Lower in CST IV |
| Bifidobacteriaceae | Gardnerella_sp | 3.466 | 0.665 | 1.2E-06 | 4.1E-05 | Enriched in CST IV |
| Bifidobacteriaceae | Gardnerella_pickettii | 5.752 | 1.111 | 1.4E-06 | 4.5E-05 | Enriched in CST IV |
| Bifidobacteriaceae | Gardnerella_sp | 4.386 | 0.855 | 1.7E-06 | 5.2E-05 | Enriched in CST IV |
| Bifidobacteriaceae | Gardnerella_sp | 4.804 | 0.943 | 2.0E-06 | 5.9E-05 | Enriched in CST IV |
| Bifidobacteriaceae | Gardnerella_sp | 4.716 | 0.932 | 2.2E-06 | 6.4E-05 | Enriched in CST IV |
| Bifidobacteriaceae | Gardnerella_vaginalis_ATCC_14019 | 2.409 | 0.500 | 5.9E-06 | 1.6E-04 | Enriched in CST IV |
| Lactobacillaceae | Leuconostoc_carnosum | -1.363 | 0.283 | 5.9E-06 | 1.6E-04 | Lower in CST IV |
| Lactobacillaceae | Lactobacillus_helveticus_CNRZ32 | -1.646 | 0.343 | 6.3E-06 | 1.7E-04 | Lower in CST IV |
| Aerococcaceae | Aerococcus_christensenii | 4.716 | 1.004 | 9.5E-06 | 2.4E-04 | Enriched in CST IV |
| Lactobacillaceae | Lactobacillus_helveticus_DPC_4571 | -2.101 | 0.453 | 1.2E-05 | 3.0E-04 | Lower in CST IV |
| Lactobacillaceae | Lactobacillus_intestinalis | -3.627 | 0.785 | 1.3E-05 | 3.1E-04 | Lower in CST IV |
| Bifidobacteriaceae | Gardnerella_sp | 5.192 | 1.137 | 1.6E-05 | 3.6E-04 | Enriched in CST IV |
| Lactobacillaceae | Lactobacillus_amylovorus_GRL1118 | -1.900 | 0.417 | 1.6E-05 | 3.6E-04 | Lower in CST IV |
| Lactobacillaceae | Lactobacillus_amylolyticus | -3.661 | 0.806 | 1.7E-05 | 3.7E-04 | Lower in CST IV |
| Bifidobacteriaceae | Gardnerella_sp | 3.731 | 0.842 | 2.7E-05 | 5.6E-04 | Enriched in CST IV |
| Bifidobacteriaceae | Gardnerella_sp | 4.254 | 0.963 | 2.8E-05 | 5.7E-04 | Enriched in CST IV |
| Bifidobacteriaceae | Gardnerella_sp | 4.898 | 1.126 | 3.6E-05 | 7.2E-04 | Enriched in CST IV |
| Lactobacillaceae | Lactobacillus_delbrueckii | -3.522 | 0.814 | 4.0E-05 | 7.5E-04 | Lower in CST IV |
| Lactobacillaceae | Companilactobacillus_paralimentarius | -1.921 | 0.444 | 3.9E-05 | 7.5E-04 | Lower in CST IV |
| Bifidobacteriaceae | Gardnerella_sp | 2.842 | 0.662 | 4.5E-05 | 8.3E-04 | Enriched in CST IV |
| Bifidobacteriaceae | Gardnerella_sp | 3.719 | 0.869 | 4.7E-05 | 8.5E-04 | Enriched in CST IV |
| Bifidobacteriaceae | Gardnerella_sp | 3.965 | 0.928 | 4.8E-05 | 8.5E-04 | Enriched in CST IV |
| Lactobacillaceae | Ligilactobacillus_saerimneri | -1.873 | 0.442 | 5.5E-05 | 9.6E-04 | Lower in CST IV |
| Bifidobacteriaceae | Gardnerella_sp | 4.184 | 0.991 | 5.8E-05 | 9.9E-04 | Enriched in CST IV |
| Bifidobacteriaceae | Gardnerella_sp | 5.020 | 1.218 | 8.5E-05 | 1.4E-03 | Enriched in CST IV |
| Bifidobacteriaceae | Gardnerella_sp | 4.458 | 1.099 | 1.1E-04 | 1.7E-03 | Enriched in CST IV |
| Bifidobacteriaceae | Gardnerella_sp | 3.703 | 0.921 | 1.2E-04 | 1.9E-03 | Enriched in CST IV |
| Bifidobacteriaceae | Gardnerella_sp | 3.200 | 0.805 | 1.4E-04 | 2.1E-03 | Enriched in CST IV |
| Lactobacillaceae | Ligilactobacillus_ruminis | -3.154 | 0.793 | 1.4E-04 | 2.1E-03 | Lower in CST IV |
| Bifidobacteriaceae | Gardnerella_sp | 4.036 | 1.029 | 1.7E-04 | 2.4E-03 | Enriched in CST IV |
| Bifidobacteriaceae | Gardnerella_sp | 3.874 | 0.989 | 1.8E-04 | 2.4E-03 | Enriched in CST IV |
| Lactobacillaceae | Limosilactobacillus_frumenti | -1.766 | 0.453 | 1.9E-04 | 2.6E-03 | Lower in CST IV |
| Lactobacillaceae | Latilactobacillus_sakei | -2.117 | 0.551 | 2.3E-04 | 3.0E-03 | Lower in CST IV |
| Oscillospiraceae | Mageeibacillus_indolicus_UPII9_5 | 3.375 | 0.881 | 2.4E-04 | 3.0E-03 | Enriched in CST IV |
| Oscillospiraceae | Mageeibacillus_indolicus | 3.375 | 0.881 | 2.4E-04 | 3.0E-03 | Enriched in CST IV |
| Lactobacillaceae | Lactobacillus_delbrueckii_subsp | -2.145 | 0.560 | 2.4E-04 | 3.0E-03 | Lower in CST IV |
| Bifidobacteriaceae | Gardnerella_sp | 3.261 | 0.854 | 2.5E-04 | 3.1E-03 | Enriched in CST IV |
| Lactobacillaceae | Lactobacillus_acidophilus | -3.273 | 0.864 | 2.8E-04 | 3.3E-03 | Lower in CST IV |
| Bifidobacteriaceae | Gardnerella_sp | 3.804 | 1.009 | 2.9E-04 | 3.5E-03 | Enriched in CST IV |
| Atopobiaceae | Fannyhessea_vaginae | 6.165 | 1.645 | 3.2E-04 | 3.6E-03 | Enriched in CST IV |
| Bifidobacteriaceae | Gardnerella_sp | 3.681 | 0.981 | 3.1E-04 | 3.6E-03 | Enriched in CST IV |
| Oscillospiraceae | Amygdalobacter_indicium | 4.203 | 1.136 | 3.7E-04 | 4.1E-03 | Enriched in CST IV |
| Lactobacillaceae | Lactobacillus_helveticus_H9 | -1.695 | 0.459 | 3.8E-04 | 4.2E-03 | Lower in CST IV |
| Bifidobacteriaceae | Gardnerella_sp | 3.002 | 0.816 | 4.0E-04 | 4.3E-03 | Enriched in CST IV |
| Lactobacillaceae | Lactobacillus_kefiranofaciens | -3.118 | 0.857 | 4.6E-04 | 4.9E-03 | Lower in CST IV |
| Bifidobacteriaceae | Gardnerella_sp | 3.154 | 0.873 | 5.0E-04 | 5.1E-03 | Enriched in CST IV |
| Bifidobacteriaceae | Gardnerella_sp | 4.511 | 1.250 | 5.1E-04 | 5.1E-03 | Enriched in CST IV |
| Bifidobacteriaceae | Gardnerella_sp | 3.398 | 0.941 | 5.0E-04 | 5.1E-03 | Enriched in CST IV |
| Lactobacillaceae | Lactobacillus_paragasseri | -3.029 | 0.846 | 5.6E-04 | 5.5E-03 | Lower in CST IV |
| Bifidobacteriaceae | Gardnerella_vaginalis_HMP9231 | 3.307 | 0.926 | 5.7E-04 | 5.6E-03 | Enriched in CST IV |
| Clostridiaceae | Clostridium_perfringens | -1.739 | 0.490 | 6.3E-04 | 5.8E-03 | Lower in CST IV |
| Bifidobacteriaceae | Gardnerella_sp | 3.696 | 1.048 | 6.7E-04 | 6.2E-03 | Enriched in CST IV |
| Lactobacillaceae | Limosilactobacillus_portuensis | -3.821 | 1.100 | 7.9E-04 | 7.1E-03 | Lower in CST IV |
| Bifidobacteriaceae | Gardnerella_sp | 3.166 | 0.916 | 8.4E-04 | 7.4E-03 | Enriched in CST IV |
| Lactobacillaceae | Limosilactobacillus_reuteri | -3.436 | 1.000 | 9.0E-04 | 7.8E-03 | Lower in CST IV |
| Bifidobacteriaceae | Gardnerella_sp | 3.554 | 1.062 | 1.2E-03 | 1.0E-02 | Enriched in CST IV |
| Lactobacillaceae | Limosilactobacillus_vaginalis | -2.152 | 0.643 | 1.2E-03 | 1.0E-02 | Lower in CST IV |
| Atopobiaceae | Lancefieldella_parvula | 1.478 | 0.453 | 1.6E-03 | 1.3E-02 | Enriched in CST IV |
| Atopobiaceae | Lancefieldella_parvula_DSM_20469 | 1.478 | 0.453 | 1.6E-03 | 1.3E-02 | Enriched in CST IV |
| Bifidobacteriaceae | Gardnerella_sp | 3.311 | 1.021 | 1.7E-03 | 1.4E-02 | Enriched in CST IV |
| Bifidobacteriaceae | Gardnerella_sp | 3.709 | 1.158 | 1.9E-03 | 1.5E-02 | Enriched in CST IV |
| Lactobacillaceae | Pediococcus_parvulus | -1.099 | 0.345 | 2.0E-03 | 1.6E-02 | Lower in CST IV |
| Bifidobacteriaceae | Gardnerella_sp | 1.370 | 0.442 | 2.6E-03 | 2.0E-02 | Enriched in CST IV |
| Bifidobacteriaceae | Gardnerella_swidsinskii | 3.706 | 1.205 | 2.8E-03 | 2.1E-02 | Enriched in CST IV |
| Bifidobacteriaceae | Gardnerella_sp | 3.424 | 1.118 | 2.9E-03 | 2.1E-02 | Enriched in CST IV |
| Lactobacillaceae | Lactobacillus_johnsonii | -2.445 | 0.801 | 3.0E-03 | 2.1E-02 | Lower in CST IV |
| Lactobacillaceae | Pediococcus_damnosus | -1.492 | 0.489 | 3.0E-03 | 2.1E-02 | Lower in CST IV |
| Bifidobacteriaceae | Gardnerella_sp | 2.647 | 0.878 | 3.3E-03 | 2.3E-02 | Enriched in CST IV |
| Bifidobacteriaceae | Gardnerella_sp | 2.207 | 0.752 | 4.2E-03 | 2.8E-02 | Enriched in CST IV |
| Bifidobacteriaceae | Gardnerella_sp | 2.075 | 0.713 | 4.5E-03 | 2.9E-02 | Enriched in CST IV |
| Bifidobacteriaceae | Gardnerella_sp | 3.050 | 1.068 | 5.3E-03 | 3.4E-02 | Enriched in CST IV |
| Bacteroidaceae | Phocaeicola_dorei | -1.651 | 0.584 | 5.8E-03 | 3.7E-02 | Lower in CST IV |
| Bifidobacteriaceae | Gardnerella_leopoldii | 2.922 | 1.038 | 6.0E-03 | 3.8E-02 | Enriched in CST IV |
| Bifidobacteriaceae | Gardnerella_vaginalis_ATCC_14018_JCM_11026 | 1.202 | 0.430 | 6.4E-03 | 4.0E-02 | Enriched in CST IV |
| Leptotrichiaceae | Sneathia_vaginalis | 2.766 | 1.007 | 7.3E-03 | 4.5E-02 | Enriched in CST IV |

**Supplementary Table 5.** Cross-validated Random Forest performance for CST IV classification using non-bacterial kingdom profiles

| Kingdom | Accuracy | Balanced accuracy | Macro F1-score | Weighted F1-score | ROC-AUC | Interpretation |
| --- | --- | --- | --- | --- | --- | --- |
| Fungi | 0.533 | 0.531 | 0.530 | 0.534 | 0.514 | Near-random / weak |
| Viruses | 0.511 | 0.501 | 0.501 | 0.509 | 0.502 | No meaningful prediction |
| Archaea | 0.554 | 0.538 | 0.534 | 0.545 | 0.560 | Weak, slightly above random |

**Supplementary Table 6.** Significant cross-kingdom associations after FDR correction

| CST | Kingdom 1 | Taxon 1 | Kingdom 2 | Taxon 2 | Spearman_r | Raw_p | FDR_q |
| --- | --- | --- | --- | --- | --- | --- | --- |
| CST I | Bacteria | Lactobacillus crispatus | Fungi | Candida | 0.672 | 0.002 | 0.028 |
| CST I | Bacteria | Lactobacillus crispatus | Fungi | Sugiyamaella | 0.832 | 0.000 | 0.001 |
| CST I | Bacteria | Lactobacillus crispatus | Fungi | Lodderomyces | 0.707 | 0.001 | 0.017 |
| CST I | Bacteria | Lactobacillus crispatus | Fungi | Australozyma | 0.847 | 0.000 | 0.001 |
| CST I | Bacteria | Lactobacillus crispatus | Fungi | Torulaspora | 0.844 | 0.000 | 0.001 |
| CST I | Bacteria | Lactobacillus crispatus | Archaea | Methanosphaera | 0.881 | 0.000 | 0.000 |
| CST I | Bacteria | Lactobacillus crispatus | Archaea | Methanosarcina | 0.628 | 0.004 | 0.049 |
| CST I | Bacteria | Lactobacillus crispatus | Archaea | Natrialba | 0.646 | 0.003 | 0.040 |
| CST I | Bacteria | Lactobacillus crispatus | Archaea | Thermococcus | 0.825 | 0.000 | 0.001 |
| CST I | Bacteria | Lactobacillus crispatus | Archaea | Sulfurisphaera | 0.749 | 0.000 | 0.009 |
| CST I | Bacteria | Lactobacillus crispatus | Archaea | Methanospirillum | 0.718 | 0.001 | 0.015 |
| CST I | Bacteria | Lactobacillus crispatus | Archaea | Archaeoglobus | 0.804 | 0.000 | 0.002 |
| CST I | Bacteria | Lactobacillus crispatus | Archaea | Halosolutus | 0.886 | 0.000 | 0.000 |
| CST I | Bacteria | Lactobacillus crispatus | Archaea | Halocatena | 0.704 | 0.001 | 0.018 |
| CST I | Bacteria | Lactobacillus crispatus | Archaea | Halomontanus | 0.739 | 0.000 | 0.011 |
| CST I | Bacteria | Lactobacillus crispatus | Viruses | Valbvirus | 0.796 | 0.000 | 0.003 |
| CST I | Bacteria | Lactobacillus crispatus | Viruses | Andhravirus | 0.789 | 0.000 | 0.003 |
| CST I | Bacteria | Lactobacillus crispatus | Viruses | Cytomegalovirus | 0.716 | 0.001 | 0.015 |
| CST I | Bacteria | Lactobacillus crispatus | Viruses | Sunrhavirus | 0.723 | 0.000 | 0.014 |
| CST I | Bacteria | Lactobacillus crispatus | Viruses | Sobemovirus | 0.711 | 0.001 | 0.016 |
| CST I | Bacteria | Lactobacillus crispatus | Viruses | Alphatorquevirus | 0.718 | 0.001 | 0.015 |
| CST I | Bacteria | Staphylococcus aureus | Fungi | Yarrowia | 0.698 | 0.001 | 0.019 |
| CST I | Bacteria | Klebsiella pneumoniae | Viruses | Sunrhavirus | 0.651 | 0.003 | 0.038 |
| CST I | Bacteria | Lactobacillus iners | Fungi | Malassezia | 0.635 | 0.003 | 0.046 |
| CST I | Bacteria | Lactobacillus iners | Archaea | Saccharolobus | 0.647 | 0.003 | 0.039 |
| CST I | Bacteria | Lactobacillus iners | Archaea | Methanosarcina | 0.800 | 0.000 | 0.003 |
| CST I | Bacteria | Lactobacillus iners | Viruses | Sunrhavirus | 0.768 | 0.000 | 0.006 |
| CST I | Bacteria | Gardnerella swidsinskii | Fungi | Lodderomyces | 0.656 | 0.002 | 0.036 |
| CST I | Bacteria | Gardnerella swidsinskii | Archaea | Thermococcus | 0.644 | 0.003 | 0.041 |
| CST I | Bacteria | Gardnerella swidsinskii | Archaea | Archaeoglobus | 0.672 | 0.002 | 0.028 |
| CST I | Bacteria | Gardnerella swidsinskii | Archaea | Halocatena | 0.753 | 0.000 | 0.009 |
| CST I | Bacteria | Gardnerella swidsinskii | Viruses | Sobemovirus | 0.802 | 0.000 | 0.003 |
| CST I | Bacteria | Lactiplantibacillus plantarum | Fungi | Lodderomyces | 0.632 | 0.004 | 0.048 |
| CST I | Bacteria | Lactiplantibacillus plantarum | Fungi | Torulaspora | 0.658 | 0.002 | 0.035 |
| CST I | Bacteria | Lactiplantibacillus plantarum | Archaea | Thermococcus | 0.711 | 0.001 | 0.016 |
| CST I | Bacteria | Lactiplantibacillus plantarum | Archaea | Halocatena | 0.630 | 0.004 | 0.049 |
| CST I | Bacteria | Lactiplantibacillus plantarum | Viruses | Andhravirus | 0.686 | 0.001 | 0.023 |
| CST I | Bacteria | Peptoniphilus equinus | Archaea | Methanobrevibacter | 0.675 | 0.002 | 0.027 |
| CST I | Bacteria | Peptoniphilus equinus | Archaea | Methanosphaera | 0.660 | 0.002 | 0.034 |
| CST I | Bacteria | Peptoniphilus equinus | Archaea | Natrialba | 0.846 | 0.000 | 0.001 |
| CST I | Bacteria | Peptoniphilus equinus | Archaea | Methanospirillum | 0.877 | 0.000 | 0.000 |
| CST I | Bacteria | Peptoniphilus equinus | Archaea | Halosolutus | 0.665 | 0.002 | 0.031 |
| CST I | Bacteria | Peptoniphilus equinus | Viruses | Caudoviricetes unclassified | 0.665 | 0.002 | 0.031 |
| CST I | Bacteria | Peptoniphilus equinus | Viruses | Rosenblumvirus | 0.644 | 0.003 | 0.041 |
| CST I | Bacteria | Peptoniphilus equinus | Viruses | Lentivirus | 0.712 | 0.001 | 0.016 |
| CST I | Bacteria | Gardnerella sp. DNF01180P | Fungi | Sugiyamaella | 0.777 | 0.000 | 0.005 |
| CST I | Bacteria | Gardnerella sp. DNF01180P | Fungi | Vairimorpha | 0.788 | 0.000 | 0.003 |
| CST I | Bacteria | Gardnerella sp. DNF01180P | Fungi | Australozyma | 0.814 | 0.000 | 0.002 |
| CST I | Bacteria | Gardnerella sp. DNF01180P | Archaea | Methanosphaera | 0.637 | 0.003 | 0.046 |
| CST I | Bacteria | Gardnerella sp. DNF01180P | Archaea | Natrialba | 0.651 | 0.003 | 0.038 |
| CST I | Bacteria | Gardnerella sp. DNF01180P | Archaea | Archaeoglobus | 0.867 | 0.000 | 0.000 |
| CST I | Bacteria | Gardnerella sp. DNF01180P | Archaea | Halosolutus | 0.642 | 0.003 | 0.042 |
| CST I | Bacteria | Gardnerella sp. DNF01180P | Viruses | Cytomegalovirus | 0.760 | 0.000 | 0.007 |
| CST I | Bacteria | Gardnerella sp. DNF01180P | Viruses | Sobemovirus | 0.730 | 0.000 | 0.013 |
| CST I | Bacteria | Escherichia coli | Fungi | Nakaseomyces | 0.714 | 0.001 | 0.015 |
| CST I | Bacteria | Escherichia coli | Fungi | Rhizoctonia | 0.688 | 0.001 | 0.023 |
| CST I | Bacteria | Escherichia coli | Fungi | Zygotorulaspora | 0.646 | 0.003 | 0.040 |
| CST I | Bacteria | Escherichia coli | Viruses | Sunrhavirus | 0.688 | 0.001 | 0.023 |
| CST I | Bacteria | Streptococcus agalactiae | Fungi | Vairimorpha | -0.702 | 0.001 | 0.018 |
| CST I | Bacteria | Streptococcus agalactiae | Archaea | Archaeoglobus | -0.642 | 0.003 | 0.042 |
| CST I | Bacteria | Varibaculum prostatecancerukia | Archaea | Natrialba | 0.686 | 0.001 | 0.023 |
| CST I | Bacteria | Varibaculum prostatecancerukia | Viruses | Rosenblumvirus | 0.653 | 0.002 | 0.038 |
| CST I | Bacteria | Facklamia hominis | Fungi | Sugiyamaella | 0.675 | 0.002 | 0.027 |
| CST I | Bacteria | Facklamia hominis | Fungi | Australozyma | 0.712 | 0.001 | 0.016 |
| CST I | Bacteria | Facklamia hominis | Archaea | Natrialba | 0.998 | 0.000 | 0.000 |
| CST I | Bacteria | Facklamia hominis | Archaea | Methanospirillum | 0.775 | 0.000 | 0.005 |
| CST I | Bacteria | Peptostreptococcus anaerobius | Fungi | Sugiyamaella | 0.670 | 0.002 | 0.029 |
| CST I | Bacteria | Peptostreptococcus anaerobius | Fungi | Australozyma | 0.714 | 0.001 | 0.015 |
| CST I | Bacteria | Peptostreptococcus anaerobius | Archaea | Natrialba | 0.972 | 0.000 | 0.000 |
| CST I | Bacteria | Peptostreptococcus anaerobius | Archaea | Methanospirillum | 0.768 | 0.000 | 0.006 |
| CST I | Bacteria | Staphylococcus pseudintermedius | Archaea | Methanosarcina | 0.628 | 0.004 | 0.049 |
| CST I | Bacteria | Staphylococcus pseudintermedius | Viruses | Sunrhavirus | 0.728 | 0.000 | 0.013 |
| CST I | Bacteria | Leuconostoc carnosum | Archaea | Sulfurisphaera | 0.747 | 0.000 | 0.009 |
| CST I | Bacteria | Leuconostoc carnosum | Archaea | Halosolutus | 0.704 | 0.001 | 0.018 |
| CST I | Bacteria | Leuconostoc carnosum | Archaea | Halomontanus | 0.700 | 0.001 | 0.019 |
| CST I | Bacteria | Leuconostoc carnosum | Viruses | Alphatorquevirus | 0.774 | 0.000 | 0.005 |
| CST I | Bacteria | Lactobacillus crispatus 2029 | Archaea | Methanosphaera | 0.633 | 0.004 | 0.047 |
| CST I | Bacteria | Lactobacillus crispatus 2029 | Archaea | Methanosarcina | 0.675 | 0.002 | 0.027 |
| CST I | Bacteria | Lactobacillus crispatus 2029 | Archaea | Sulfurisphaera | 0.637 | 0.003 | 0.046 |
| CST I | Bacteria | Lactobacillus crispatus 2029 | Viruses | Sunrhavirus | 0.730 | 0.000 | 0.013 |
| CST I | Bacteria | Lactobacillus amylovorus | Viruses | Sunrhavirus | 0.721 | 0.000 | 0.014 |
| CST I | Fungi | Malassezia | Archaea | Saccharolobus | 0.904 | 0.000 | 0.000 |
| CST I | Fungi | Malassezia | Viruses | Andhravirus | 0.651 | 0.003 | 0.038 |
| CST I | Fungi | Malassezia | Viruses | Sunrhavirus | 0.684 | 0.001 | 0.024 |
| CST I | Fungi | Candida | Archaea | Thermococcus | 0.672 | 0.002 | 0.028 |
| CST I | Fungi | Candida | Archaea | Halosolutus | 0.709 | 0.001 | 0.016 |
| CST I | Fungi | Henningerozyma | Viruses | Kagamiyamavirus | 0.749 | 0.000 | 0.009 |
| CST I | Fungi | Henningerozyma | Viruses | Nodensvirus | 0.749 | 0.000 | 0.009 |
| CST I | Fungi | Sugiyamaella | Archaea | Methanosphaera | 0.716 | 0.001 | 0.015 |
| CST I | Fungi | Sugiyamaella | Archaea | Natrialba | 0.700 | 0.001 | 0.019 |
| CST I | Fungi | Sugiyamaella | Archaea | Thermococcus | 0.635 | 0.003 | 0.046 |
| CST I | Fungi | Sugiyamaella | Archaea | Archaeoglobus | 0.681 | 0.001 | 0.025 |
| CST I | Fungi | Sugiyamaella | Archaea | Halosolutus | 0.911 | 0.000 | 0.000 |
| CST I | Fungi | Sugiyamaella | Viruses | Valbvirus | 0.718 | 0.001 | 0.015 |
| CST I | Fungi | Sugiyamaella | Viruses | Cytomegalovirus | 0.789 | 0.000 | 0.003 |
| CST I | Fungi | Sugiyamaella | Viruses | Alphatorquevirus | 0.630 | 0.004 | 0.049 |
| CST I | Fungi | Nakaseomyces | Archaea | Sulfurisphaera | 0.725 | 0.000 | 0.013 |
| CST I | Fungi | Nakaseomyces | Archaea | Halomontanus | 0.704 | 0.001 | 0.018 |
| CST I | Fungi | Nakaseomyces | Viruses | Sunrhavirus | 0.698 | 0.001 | 0.019 |
| CST I | Fungi | Nakaseomyces | Viruses | Alphatorquevirus | 0.693 | 0.001 | 0.021 |
| CST I | Fungi | Vairimorpha | Archaea | Methanosphaera | 0.646 | 0.003 | 0.040 |
| CST I | Fungi | Vairimorpha | Archaea | Archaeoglobus | 0.660 | 0.002 | 0.034 |
| CST I | Fungi | Psilocybe | Archaea | Geoglobus | 0.651 | 0.003 | 0.038 |
| CST I | Fungi | Pichia | Archaea | Saccharolobus | 0.921 | 0.000 | 0.000 |
| CST I | Fungi | Pichia | Viruses | Sunrhavirus | 0.651 | 0.003 | 0.038 |
| CST I | Fungi | Lodderomyces | Archaea | Methanosphaera | 0.668 | 0.002 | 0.029 |
| CST I | Fungi | Lodderomyces | Archaea | Thermococcus | 0.695 | 0.001 | 0.020 |
| CST I | Fungi | Lodderomyces | Archaea | Halocatena | 0.905 | 0.000 | 0.000 |
| CST I | Fungi | Lodderomyces | Viruses | Andhravirus | 0.695 | 0.001 | 0.020 |
| CST I | Fungi | Lodderomyces | Viruses | Sobemovirus | 0.633 | 0.004 | 0.047 |
| CST I | Fungi | Australozyma | Archaea | Methanosphaera | 0.742 | 0.000 | 0.010 |
| CST I | Fungi | Australozyma | Archaea | Natrialba | 0.739 | 0.000 | 0.011 |
| CST I | Fungi | Australozyma | Archaea | Thermococcus | 0.751 | 0.000 | 0.009 |
| CST I | Fungi | Australozyma | Archaea | Archaeoglobus | 0.702 | 0.001 | 0.018 |
| CST I | Fungi | Australozyma | Archaea | Halosolutus | 0.749 | 0.000 | 0.009 |
| CST I | Fungi | Australozyma | Viruses | Valbvirus | 0.798 | 0.000 | 0.003 |
| CST I | Fungi | Australozyma | Viruses | Andhravirus | 0.653 | 0.002 | 0.038 |
| CST I | Fungi | Torulaspora | Archaea | Methanosphaera | 0.725 | 0.000 | 0.013 |
| CST I | Fungi | Torulaspora | Archaea | Thermococcus | 0.963 | 0.000 | 0.000 |
| CST I | Fungi | Torulaspora | Archaea | Archaeoglobus | 0.725 | 0.000 | 0.013 |
| CST I | Fungi | Torulaspora | Archaea | Halosolutus | 0.730 | 0.000 | 0.013 |
| CST I | Fungi | Torulaspora | Archaea | Halocatena | 0.789 | 0.000 | 0.003 |
| CST I | Fungi | Torulaspora | Viruses | Andhravirus | 0.647 | 0.003 | 0.039 |
| CST I | Fungi | Torulaspora | Viruses | Kagamiyamavirus | 0.733 | 0.000 | 0.012 |
| CST I | Fungi | Torulaspora | Viruses | Nodensvirus | 0.733 | 0.000 | 0.012 |
| CST I | Fungi | Zygotorulaspora | Archaea | Sulfurisphaera | 0.649 | 0.003 | 0.039 |
| CST I | Fungi | Zygotorulaspora | Archaea | Halomontanus | 0.628 | 0.004 | 0.049 |
| CST I | Fungi | Zygotorulaspora | Viruses | Sunrhavirus | 0.682 | 0.001 | 0.024 |
| CST I | Fungi | Akanthomyces | Archaea | Sulfurisphaera | 0.679 | 0.001 | 0.025 |
| CST I | Fungi | Akanthomyces | Archaea | Halomontanus | 0.658 | 0.002 | 0.035 |
| CST I | Fungi | Akanthomyces | Viruses | Sunrhavirus | 0.712 | 0.001 | 0.016 |
| CST I | Fungi | Akanthomyces | Viruses | Alphatorquevirus | 0.732 | 0.000 | 0.013 |
| CST I | Fungi | Kluyveromyces | Archaea | Sulfurisphaera | 0.756 | 0.000 | 0.008 |
| CST I | Fungi | Kluyveromyces | Archaea | Geoglobus | 1.000 | 0.000 | 0.000 |
| CST I | Fungi | Kluyveromyces | Archaea | Halomontanus | 0.735 | 0.000 | 0.012 |
| CST I | Fungi | Kluyveromyces | Viruses | Rubivirus | 0.825 | 0.000 | 0.001 |
| CST I | Fungi | Kluyveromyces | Viruses | Alphatorquevirus | 0.725 | 0.000 | 0.013 |
| CST I | Archaea | Methanobrevibacter | Viruses | Alphatorquevirus | 0.628 | 0.004 | 0.049 |
| CST I | Archaea | Saccharolobus | Viruses | Andhravirus | 0.628 | 0.004 | 0.049 |
| CST I | Archaea | Saccharolobus | Viruses | Sunrhavirus | 0.679 | 0.001 | 0.025 |
| CST I | Archaea | Methanosphaera | Viruses | Valbvirus | 0.709 | 0.001 | 0.016 |
| CST I | Archaea | Methanosphaera | Viruses | Andhravirus | 0.672 | 0.002 | 0.028 |
| CST I | Archaea | Methanosphaera | Viruses | Cytomegalovirus | 0.653 | 0.002 | 0.038 |
| CST I | Archaea | Methanosphaera | Viruses | Rubivirus | 0.630 | 0.004 | 0.049 |
| CST I | Archaea | Methanosarcina | Viruses | Sunrhavirus | 0.902 | 0.000 | 0.000 |
| CST I | Archaea | Methanosarcina | Viruses | Lentivirus | 0.635 | 0.003 | 0.046 |
| CST I | Archaea | Thermococcus | Viruses | Kagamiyamavirus | 0.686 | 0.001 | 0.023 |
| CST I | Archaea | Thermococcus | Viruses | Nodensvirus | 0.686 | 0.001 | 0.023 |
| CST I | Archaea | Sulfurisphaera | Viruses | Rubivirus | 0.668 | 0.002 | 0.029 |
| CST I | Archaea | Sulfurisphaera | Viruses | Kagamiyamavirus | 0.811 | 0.000 | 0.002 |
| CST I | Archaea | Sulfurisphaera | Viruses | Alphatorquevirus | 0.958 | 0.000 | 0.000 |
| CST I | Archaea | Sulfurisphaera | Viruses | Nodensvirus | 0.811 | 0.000 | 0.002 |
| CST I | Archaea | Geoglobus | Viruses | Rubivirus | 0.825 | 0.000 | 0.001 |
| CST I | Archaea | Geoglobus | Viruses | Alphatorquevirus | 0.725 | 0.000 | 0.013 |
| CST I | Archaea | Methanospirillum | Viruses | Caudoviricetes unclassified | 0.651 | 0.003 | 0.038 |
| CST I | Archaea | Methanospirillum | Viruses | Rosenblumvirus | 0.744 | 0.000 | 0.010 |
| CST I | Archaea | Archaeoglobus | Viruses | Cytomegalovirus | 0.860 | 0.000 | 0.000 |
| CST I | Archaea | Archaeoglobus | Viruses | Sobemovirus | 0.828 | 0.000 | 0.001 |
| CST I | Archaea | Halosolutus | Viruses | Valbvirus | 0.688 | 0.001 | 0.023 |
| CST I | Archaea | Halosolutus | Viruses | Cytomegalovirus | 0.884 | 0.000 | 0.000 |
| CST I | Archaea | Halosolutus | Viruses | Alphatorquevirus | 0.681 | 0.001 | 0.025 |
| CST I | Archaea | Halocatena | Viruses | Valbvirus | 0.633 | 0.004 | 0.047 |
| CST I | Archaea | Halocatena | Viruses | Andhravirus | 0.719 | 0.001 | 0.015 |
| CST I | Archaea | Halocatena | Viruses | Sobemovirus | 0.684 | 0.001 | 0.024 |
| CST I | Archaea | Halomontanus | Viruses | Rubivirus | 0.647 | 0.003 | 0.039 |
| CST I | Archaea | Halomontanus | Viruses | Kagamiyamavirus | 0.789 | 0.000 | 0.003 |
| CST I | Archaea | Halomontanus | Viruses | Alphatorquevirus | 0.937 | 0.000 | 0.000 |
| CST I | Archaea | Halomontanus | Viruses | Nodensvirus | 0.789 | 0.000 | 0.003 |
| CST III | Bacteria | Lactobacillus iners | Fungi | Zygotorulaspora | 0.675 | 0.001 | 0.028 |
| CST III | Bacteria | Lactobacillus iners | Fungi | Encephalitozoon | 0.645 | 0.001 | 0.040 |
| CST III | Bacteria | Lactobacillus iners | Fungi | Remersonia | 0.799 | 0.000 | 0.003 |
| CST III | Bacteria | Lactobacillus iners | Archaea | Methanocella | 0.663 | 0.001 | 0.030 |
| CST III | Bacteria | Lactobacillus iners | Archaea | Natronorarus | 0.750 | 0.000 | 0.009 |
| CST III | Bacteria | Lactobacillus iners | Archaea | Halovivax | 0.748 | 0.000 | 0.009 |
| CST III | Bacteria | Lactobacillus iners | Viruses | Andhravirus | 0.762 | 0.000 | 0.009 |
| CST III | Bacteria | Lactobacillus iners | Viruses | Valbvirus | 0.653 | 0.001 | 0.036 |
| CST III | Bacteria | Aerococcus christensenii | Viruses | Sunrhavirus | 0.750 | 0.000 | 0.009 |
| CST III | Bacteria | Lactobacillus jensenii | Fungi | Akanthomyces | 0.694 | 0.000 | 0.019 |
| CST III | Bacteria | Parvimonas parva | Fungi | Vairimorpha | 0.694 | 0.000 | 0.019 |
| CST III | Bacteria | Parvimonas parva | Fungi | Malassezia | 0.719 | 0.000 | 0.012 |
| CST III | Bacteria | Parvimonas parva | Fungi | Remersonia | 0.631 | 0.002 | 0.047 |
| CST III | Bacteria | Parvimonas parva | Archaea | Natronorarus | 0.854 | 0.000 | 0.000 |
| CST III | Bacteria | Klebsiella pneumoniae | Fungi | Yarrowia | 0.693 | 0.000 | 0.019 |
| CST III | Bacteria | Klebsiella pneumoniae | Viruses | Sunrhavirus | 0.724 | 0.000 | 0.011 |
| CST III | Bacteria | Escherichia coli | Fungi | Yarrowia | 0.798 | 0.000 | 0.003 |
| CST III | Bacteria | Escherichia coli | Fungi | Akanthomyces | 0.665 | 0.001 | 0.030 |
| CST III | Bacteria | Helcococcus ovis | Fungi | Saccharomyces | 0.709 | 0.000 | 0.015 |
| CST III | Bacteria | Helcococcus ovis | Archaea | Halovivax | 0.735 | 0.000 | 0.009 |
| CST III | Bacteria | Streptococcus anginosus | Fungi | Lodderomyces | 0.744 | 0.000 | 0.009 |
| CST III | Bacteria | Companilactobacillus pabuli | Fungi | Zygotorulaspora | 0.634 | 0.002 | 0.044 |
| CST III | Bacteria | Companilactobacillus pabuli | Fungi | Vairimorpha | 0.645 | 0.001 | 0.040 |
| CST III | Bacteria | Companilactobacillus pabuli | Fungi | Remersonia | 0.644 | 0.001 | 0.040 |
| CST III | Bacteria | Companilactobacillus pabuli | Archaea | Natronorarus | 0.670 | 0.001 | 0.029 |
| CST III | Bacteria | Companilactobacillus pabuli | Viruses | Andhravirus | 0.665 | 0.001 | 0.030 |
| CST III | Bacteria | Companilactobacillus pabuli | Viruses | Valbvirus | 0.697 | 0.000 | 0.019 |
| CST III | Bacteria | Gardnerella sp. KA01001 | Viruses | Moineauvirus | 0.724 | 0.000 | 0.011 |
| CST III | Bacteria | Tuanshanicoccus yangjingiae | Fungi | Vairimorpha | 0.637 | 0.001 | 0.042 |
| CST III | Bacteria | Tuanshanicoccus yangjingiae | Fungi | Encephalitozoon | 0.645 | 0.001 | 0.040 |
| CST III | Bacteria | Tuanshanicoccus yangjingiae | Archaea | Natronorarus | 0.710 | 0.000 | 0.015 |
| CST III | Fungi | Zygotorulaspora | Archaea | Natronorarus | 0.659 | 0.001 | 0.033 |
| CST III | Fungi | Zygotorulaspora | Viruses | Andhravirus | 0.642 | 0.001 | 0.041 |
| CST III | Fungi | Zygotorulaspora | Viruses | Rosenblumvirus | 0.677 | 0.001 | 0.028 |
| CST III | Fungi | Encephalitozoon | Archaea | Natronorarus | 0.743 | 0.000 | 0.009 |
| CST III | Fungi | Pichia | Archaea | Methanocella | 0.640 | 0.001 | 0.042 |
| CST III | Fungi | Remersonia | Archaea | Methanocella | 0.735 | 0.000 | 0.009 |
| CST III | Fungi | Remersonia | Archaea | Natronorarus | 0.666 | 0.001 | 0.030 |
| CST III | Fungi | Remersonia | Viruses | Andhravirus | 0.639 | 0.001 | 0.042 |
| CST III | Fungi | Rhizoctonia | Archaea | Halovivax | 0.770 | 0.000 | 0.008 |
| CST III | Fungi | Saccharomyces | Archaea | Halovivax | 0.674 | 0.001 | 0.028 |
| CST III | Fungi | Saccharomyces | Archaea | Thermococcus | 0.736 | 0.000 | 0.009 |
| CST IV | Bacteria | Bifidobacterium breve | Archaea | Nitrosopumilus | 0.604 | 0.000 | 0.000 |
| CST IV | Bacteria | Gardnerella sp. DNF01195 | Archaea | Methanobacterium | -0.603 | 0.000 | 0.000 |
| CST IV | Bacteria | Bifidobacterium bifidum | Fungi | Kluyveromyces | 0.641 | 0.000 | 0.000 |
| CST IV | Fungi | Rhizoctonia | Viruses | Lentivirus | 0.606 | 0.000 | 0.000 |
| CST IV | Fungi | Remersonia | Archaea | Nitrosopumilus | 0.608 | 0.000 | 0.000 |
| CST IV | Fungi | Remersonia | Viruses | Lentivirus | 0.641 | 0.000 | 0.000 |
| CST IV | Fungi | Kluyveromyces | Viruses | Efquatrovirus | 0.675 | 0.000 | 0.000 |
| CST IV | Fungi | Yarrowia | Viruses | Sunrhavirus | 0.740 | 0.000 | 0.000 |
| CST IV | Fungi | Lodderomyces | Archaea | Nitrosopumilus | 0.824 | 0.000 | 0.000 |
| CST IV | Fungi | Lodderomyces | Archaea | Saccharolobus | 0.670 | 0.000 | 0.000 |
| CST IV | Fungi | Lodderomyces | Viruses | Efquatrovirus | 0.608 | 0.000 | 0.000 |
| CST IV | Fungi | Lodderomyces | Viruses | Lentivirus | 0.685 | 0.000 | 0.000 |
| CST IV | Fungi | Henningerozyma | Archaea | Nitrosopumilus | 0.697 | 0.000 | 0.000 |
| CST IV | Fungi | Henningerozyma | Viruses | Gammaretrovirus | 0.702 | 0.000 | 0.000 |
| CST IV | Fungi | Henningerozyma | Viruses | Sunrhavirus | 0.613 | 0.000 | 0.000 |
| CST IV | Fungi | Henningerozyma | Viruses | Lentivirus | 0.704 | 0.000 | 0.000 |
| CST IV | Archaea | Acidianus | Viruses | Lentivirus | 0.603 | 0.000 | 0.000 |
| CST IV | Archaea | Nitrosopumilus | Viruses | Cytomegalovirus | 0.619 | 0.000 | 0.000 |
| CST IV | Archaea | Nitrosopumilus | Viruses | Efquatrovirus | 0.690 | 0.000 | 0.000 |
| CST IV | Archaea | Nitrosopumilus | Viruses | Lentivirus | 0.741 | 0.000 | 0.000 |
| CST IV | Archaea | Saccharolobus | Viruses | Cytomegalovirus | 0.638 | 0.000 | 0.000 |

**Supplementary Table 7. Significant MaAsLin2 pathway associations with CST group.**
Table includes pathway name, CST comparison, coefficient, standard error, p-value, q-value, and prevalence across samples.

| feature | value | coef | stderr | pval | qval |
| --- | --- | --- | --- | --- | --- |
| Carotenoid biosynthesis | CST_IV | -1.591 | 0.167 | 0.000 | 0.000 |
| Acarbose and validamycin biosynthesis | CST_IV | 2.546 | 0.285 | 0.000 | 0.000 |
| Biosynthesis of vancomycin group antibiotics | CST_IV | 2.459 | 0.312 | 0.000 | 0.000 |
| Polyketide sugar unit biosynthesis | CST_IV | 2.664 | 0.376 | 0.000 | 0.000 |
| Carbapenem biosynthesis | CST_IV | 2.146 | 0.315 | 0.000 | 0.000 |
| Phosphonate and phosphinate metabolism | CST_IV | 2.067 | 0.323 | 0.000 | 0.000 |
| Phenylalanine tyrosine and tryptophan biosynthesis | CST_IV | 2.287 | 0.367 | 0.000 | 0.000 |
| Flagellar assembly | CST_III | 2.704 | 0.445 | 0.000 | 0.000 |
| Ethylbenzene degradation | CST_IV | 1.608 | 0.268 | 0.000 | 0.000 |
| Carotenoid biosynthesis | CST_III | -1.133 | 0.195 | 0.000 | 0.000 |
| Streptomycin biosynthesis | CST_IV | 1.505 | 0.266 | 0.000 | 0.000 |
| Peroxisome | CST_IV | 0.846 | 0.152 | 0.000 | 0.000 |
| Phenylalanine metabolism | CST_IV | 1.135 | 0.205 | 0.000 | 0.000 |
| Biosynthesis of ansamycins | CST_IV | 1.837 | 0.333 | 0.000 | 0.000 |
| Riboflavin metabolism | CST_IV | -1.796 | 0.328 | 0.000 | 0.000 |
| Bacterial chemotaxis | CST_III | 2.188 | 0.432 | 0.000 | 0.000 |
| Dioxin degradation | CST_IV | -1.470 | 0.290 | 0.000 | 0.000 |
| Monobactam biosynthesis | CST_III | -2.521 | 0.502 | 0.000 | 0.000 |
| Bacterial chemotaxis | CST_IV | 1.815 | 0.371 | 0.000 | 0.000 |
| Histidine metabolism | CST_IV | 1.727 | 0.365 | 0.000 | 0.000 |
| Xylene degradation | CST_IV | -1.400 | 0.302 | 0.000 | 0.000 |
| Lysine degradation | CST_IV | -0.561 | 0.122 | 0.000 | 0.000 |
| Styrene degradation | CST_III | 1.667 | 0.376 | 0.000 | 0.001 |
| Insulin resistance | CST_IV | 0.444 | 0.101 | 0.000 | 0.001 |
| Ascorbate and aldarate metabolism | CST_III | -2.068 | 0.472 | 0.000 | 0.001 |
| Sulfur metabolism | CST_IV | 1.307 | 0.301 | 0.000 | 0.001 |
| Porphyrin metabolism | CST_IV | 1.031 | 0.238 | 0.000 | 0.001 |
| Longevity regulating pathway multiple species | CST_IV | 1.199 | 0.280 | 0.000 | 0.001 |
| Phenylpropanoid biosynthesis | CST_IV | 1.937 | 0.481 | 0.000 | 0.003 |
| Novobiocin biosynthesis | CST_IV | 1.251 | 0.313 | 0.000 | 0.003 |
| Staphylococcus aureus infection | CST_IV | -0.337 | 0.085 | 0.000 | 0.004 |
| Dioxin degradation | CST_III | -1.341 | 0.338 | 0.000 | 0.004 |
| GABAergic synapse | CST_IV | 0.603 | 0.153 | 0.000 | 0.004 |
| Secondary bile acid biosynthesis | CST_IV | -1.128 | 0.289 | 0.000 | 0.004 |
| Ferroptosis | CST_IV | 1.106 | 0.284 | 0.000 | 0.004 |
| Sphingolipid metabolism | CST_III | -0.863 | 0.222 | 0.000 | 0.004 |
| Neomycin kanamycin and gentamicin biosynthesis | CST_IV | 1.435 | 0.374 | 0.000 | 0.005 |
| Xylene degradation | CST_III | -1.341 | 0.352 | 0.000 | 0.005 |
| Fatty acid biosynthesis | CST_IV | 0.843 | 0.225 | 0.000 | 0.006 |
| Valine leucine and isoleucine degradation | CST_IV | 0.625 | 0.178 | 0.001 | 0.013 |
| Acarbose and validamycin biosynthesis | CST_III | 1.157 | 0.332 | 0.001 | 0.015 |
| C5 Branched dibasic acid metabolism | CST_III | -1.864 | 0.537 | 0.001 | 0.015 |
| Secondary bile acid biosynthesis | CST_III | -1.165 | 0.336 | 0.001 | 0.015 |
| Isoquinoline alkaloid biosynthesis | CST_IV | 0.688 | 0.200 | 0.001 | 0.015 |
| alpha Linolenic acid metabolism | CST_IV | 0.928 | 0.270 | 0.001 | 0.015 |
| Primary bile acid biosynthesis | CST_IV | -1.046 | 0.319 | 0.001 | 0.026 |
| Benzoate degradation | CST_IV | -1.114 | 0.345 | 0.002 | 0.028 |
| Valine leucine and isoleucine biosynthesis | CST_IV | 1.267 | 0.392 | 0.002 | 0.028 |
| Primary bile acid biosynthesis | CST_III | -1.207 | 0.372 | 0.002 | 0.028 |
| Ubiquinone and other terpenoid quinone biosynthesis | CST_III | -1.516 | 0.473 | 0.002 | 0.029 |
| Lysine biosynthesis | CST_IV | -0.855 | 0.268 | 0.002 | 0.030 |
| Nonribosomal peptide structures | CST_IV | -0.795 | 0.250 | 0.002 | 0.030 |
| Tropane piperidine and pyridine alkaloid biosynthesis | CST_III | -0.976 | 0.308 | 0.002 | 0.031 |
| Renin angiotensin system | CST_IV | 1.058 | 0.333 | 0.002 | 0.031 |
| Glycerolipid metabolism | CST_IV | -0.642 | 0.204 | 0.002 | 0.032 |
| Aminobenzoate degradation | CST_IV | -1.090 | 0.350 | 0.002 | 0.035 |
| Two component system | CST_IV | -0.903 | 0.291 | 0.003 | 0.035 |
| Phenylalanine metabolism | CST_III | 0.741 | 0.239 | 0.003 | 0.035 |
| Pancreatic secretion | CST_IV | -0.770 | 0.249 | 0.003 | 0.035 |
| Fat digestion and absorption | CST_IV | -1.011 | 0.329 | 0.003 | 0.037 |
| Lysine biosynthesis | CST_III | -0.956 | 0.312 | 0.003 | 0.038 |
| Nitrogen metabolism | CST_IV | 0.620 | 0.206 | 0.003 | 0.043 |
| PPAR signaling pathway | CST_IV | 0.516 | 0.175 | 0.004 | 0.049 |
| Ascorbate and aldarate metabolism | CST_IV | -1.196 | 0.405 | 0.004 | 0.049 |

Supplementary Figure 1. Family- and species-level archaeal composition stratified by CST group

Supplementary Figure 2. Family- and species-level fungal composition stratified by CST group

**Supplementary Figure 3**. **CST-associated bacterial taxa identified by MaAsLin2.** Heatmap showing bacterial family- and species-level taxa significantly associated with CST group membership based on MaAsLin2 analysis. CST I was used as the reference group. The colour scale represents −log10(FDR q-value) multiplied by the sign of the regression coefficient; red indicates enrichment in the comparison group relative to CST I, whereas blue indicates lower abundance relative to CST I. Only taxa with FDR-adjusted q < 0.05 are shown.

**Supplementary Figure 4. Alpha- and beta-diversity of archaeal, viral, and fungal communities across CSTs.** Alpha- and beta-diversity analyses were performed separately for archaea, viruses, and fungi across CST I, CST III, and CST IV. Community structure was assessed using Bray–Curtis PCoA with PERMANOVA and PERMDISP testing, while alpha diversity was evaluated using Observed richness, Shannon diversity, and Pielou evenness. No significant CST-associated differences were observed for archaeal, viral, or fungal alpha diversity, and beta-diversity separation was non-significant or borderline, indicating that CST stratification was primarily driven by bacterial community structure rather than consistent diversity shifts in non-bacterial kingdoms.

**Supplementary Figure 5. Exploratory taxon-level patterns across non-bacterial kingdoms by CST.** Pairwise comparisons are shown for CST I versus CST III, CST I versus CST IV, and CST III versus CST IV across fungi, archaea, and viruses. Bars represent Cohen’s d effect sizes, with positive values indicating enrichment in the first CST group and negative values indicating enrichment in the second CST group. Only taxa passing |d| > 0.25 and FDR q < 0.05 are shown. These analyses were considered exploratory because global alpha-diversity differences were not significant and beta-diversity findings were non-significant or borderline.

|  |  |
| --- | --- |
|  |  |

**Supplementary Figure 6.** **Permutation importance of non-bacterial taxa in Random Forest classification of CST IV.** Permutation-based feature importance from exploratory Random Forest models using archaeal, fungal, and viral profiles to distinguish Lactobacillus-dominated communities from CST IV/non-Lactobacillus-dominated communities. Overall model performance was weak, so taxa importance should be interpreted cautiously and as exploratory rather than as evidence of robust predictors.

**Supplementary Figure 7.** **Simplified cross-kingdom association networks by CST.** Simplified networks showing the top connected taxa involved in significant cross-kingdom associations within each CST. Node color indicates kingdom, and edge color indicates positive or negative association. These networks are exploratory and represent co-variation rather than direct ecological interactions.

**Supplementary Figure 8.** **Ranked significance of CST-associated functional pathways.** Pathways are ranked according to raw p-value from pathway-level CST association testing. The dashed red line indicates p = 0.05.

**Supplementary Figure 9. Heatmap of top CST-associated functional pathways.**
Heatmap showing the top CST-associated pathways across samples. Rows represent samples annotated by CST group, and columns represent selected functional pathways. Color intensity reflects scaled pathway abundance.
